# JA‐Mediated Regulation of Amino Acid Homeostasis Adjusts Metabolic Flux and Enhances Spider Mite Tolerance via the SlJAZ8‐SlWRKY57‐SlAVT6s Module in Tomato

**DOI:** 10.1002/advs.202416717

**Published:** 2025-06-10

**Authors:** Yingchen Hao, Xiaolong Wang, Langchen Guo, Lijun Xiang, Enxi Luo, Peng Cao, Penghui Liu, Yue Zhong, Chun Li, Jun Lai, Jun Yang, Shouchuang Wang

**Affiliations:** ^1^ National Key Laboratory for Tropical Crop Breeding School of Breeding and Multiplication(Sanya Institute of Breeding and Multiplication) Hainan University Sanya Hainan 572025 China; ^2^ National Key Laboratory for Tropical Crop Breeding College of Tropical Agriculture and Forestry Hainan University Sanya Hainan 572025 China; ^3^ Collaborative Innovation Center of Nanfan and High‐Efficiency Tropical Agriculture Hainan University Haikou 572208 China; ^4^ Yazhouwan National Laboratory (YNL) Sanya 572025 China

**Keywords:** amino acid homeostasis, JA signaling, secondary metabolism, spider mite tolerance, vacuole amino acid transporter

## Abstract

A crucial strategy employed by plants to enhance insect resistance is allocating amino acids into secondary metabolic pathways, ensuring the synthesis of specialized metabolites that confer resistance. The storage and redistribution of amino acids primarily occur in vacuole; therefore, transport mechanisms must exist to facilitate the directed extravasation of amino acids from vacuole to cytosol and feed them into secondary metabolism in response to stress. However, the specific amino acid transporter located in the vacuole responsible for amino acid distribution remains unclear. Here, we identify two tomato vacuolar amino acid transporters, SlAVT6A and SlAVT6B. SlAVT6A functions as the primary exporter, while SlAVT6B modulates transport capacity through SlAVT6A/SlAVT6B heterodimer formation. This system redirects amino acids to boost trichome density, terpene accumulation, and gibberellin synthesis, thereby strengthening defense against spider mites. Furthermore, SlWRKY57 coordinates both transporters by forming a complex with SlJAZ8, linking jasmonic acid (JA) signaling to amino acid homeostasis through metabolic reprogramming from primary to specialized pathways. The findings reveal a SlJAZ8‐SlWRKY57‐SlAVT6A/SlAVT6B module that enhances growth and resistance by allocating amino acid to secondary metabolic pathways, offering insights for improving resistance in metabolic‐assisted breeding.

## Graphical abstract

This study elucidates a novel SlJAZ8‐SlWRKY57‐SlAVT6A/SlAVT6B module that effectively channels metabolic flux from primary to secondary metabolism, impacting plant growth, development, and resistance. Two novel vacuolar amino acid exporters, SlAVT6A and SlAVT6B, fine‐tunes trichomes, terpenes, and gibberellins, bolstering spider mite resistance. This mechanistic link between amino acid homeostasis and secondary metabolism unveils strategies for engineering crops with enhanced resistance.

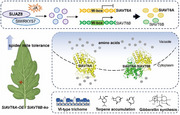

## 1 Introduction

Plants have evolved sophisticated and complex metabolic mechanisms to detect and combat various types of environments. Normally, Amino acid homeostasis exerts significant influence on plant growth and development through various pathways, including protein synthesis, nitrogen uptake and utilization, and photosynthesis.^[^ ^1^, ^2^, ^3^ ^]^ Additionally, Amino acids and their derivatives play crucial roles in synthesizing a range of complex specialized metabolites, including phytohormones, flavonoids, polyamines, glucosides, and acyl sugars, which confer resistance to stress responses.^[^ ^4^, ^5^ ^]^ The pathways responsible for amino acid metabolism are well understood, the molecular regulatory network by which amino acids affect plant growth, development, and resistance responses are still largely elusive. Thus, analyzing the molecular mechanisms of amino acid metabolic flow adjustments could help mitigate the adverse effects of stressful environments and improve plant growth and development.

Amino acid catabolism and reallocation in plants is a complex process involving the coordinated action of multiple organelles and tissues, requiring transmembrane transport to distribute amino acids across various organelles.^[^ ^5^, ^6^ ^]^ Amino acid transport, both within and between cells, is facilitated by specialized proteins known as amino acid transporters. These transporters exhibit distinct affinity and specificity for various amino acids, playing a significant role in plant growth and development.^[^ ^4^ ^]^ Vacuoles are the primary intracellular repositories for excess amino acids and are essential in maintaining cytosolic amino acid homeostasis and responding to metabolic demands.^[^ ^6^ ^]^ Amino acid vacuolar transporters (AVTs), which belong to the amino acid transporter family (ATF), are responsible for amino acid exchange between cytoplasm and vacuoles, thereby ensuring adequate allocation across cellular organelles to maintain cytosolic homeostasis and other metabolic functions.^[^ ^7^ ^]^ AVTs, categorized as members of the amino acid transporter‐like (ATL) family with further subdivision into aromatic and neutral amino acid transporter (ANT) subtypes, have been functionally characterized through cloning studies in *Arabidopsis thaliana* and *Oryza sativa*.^[^ ^8^, ^9^ ^]^ AtANT1 and AtAvt3A‐C transport aromatic and neutral amino acids, as well as auxin, although their physiological significance has not been fully elucidated.^[^ ^9^ ^]^ Studies in rice suggests that OsATL6 is involved in the temporary storage of glutamine in root vacuoles under NH_4_^+^‐rich conditions.^[^ ^8^ ^]^ Furthermore, OsATL14 has been linked to seed hull color changes that occurred during rice domestication,^[^ ^10^ ^]^ while OsATL15 is associated with thiamethoxam (THX) uptake and distribution, conferring resistance to the brown planthopper.^[^ ^11^ ^]^ Based on proteomic identification, SlCAT9 has been identified as amino acid exchanger for tonoplast, which can substantially increase the amino acid content in ripe fruit.^[^ ^12^ ^]^ However, theses researches have focused on the identification of transport activity and their effects on plant growth, while the molecular mechanisms underlying the dynamic changes of amino acids within the vacuole remain unclear.

Insect herbivory poses a major threat to global crop productivity. Spider mites have the capacity to kill or inflict damage on more than 1100 plant species, causing chlorotic leaf surface and even shedding, which seriously affecting the growth and yield of plants. The high fecundity of these pests drives rapid population growth that can have devastating consequences and as such pose a significant agricultural threat.^[^ ^4^ ^]^ Plants have evolved a variety of defensive strategies against herbivores, including morphological adaptations and enhanced production of specialized metabolites or defense‐associated proteins.^[^ ^13^ ^]^ For example, trichomes and the metabolites they secrete, such as δ‐elemene, caryophyllene, and humulene, serve as barriers to spider mite infestation.^[^ ^14^ ^]^ Additionally, plant hormones significantly influence plant tolerance to spider mite infestation.^[^ ^4^ ^]^ Notably, jasmonic acid (JA), a central regulator of plant immunity against herbivores, activates the biosynthesis of defensive compounds such as proteinase inhibitors, alkaloids, and volatile organic compounds, which directly impair insect feeding or attract natural enemies of herbivores.^[^ ^15^ ^]^ For instance, in rice, JA orchestrates resistance against the brown planthopper (BPH) by inducing the accumulation of sakuranetin, a flavonoid phytoalexin with potent insecticidal propertie.^[^ ^16^, ^17^ ^]^ Additionally, tryptophan is a key precursor for JA biosynthesis itself, while phenylalanine fuels the production of phenolic compounds and lignin, which strengthen physical barriers against herbivory.^[^ ^18^, ^19^ ^]^ In tomato, impaired JA synthesis or perception disrupts the activation of defense‐related genes, rendering plants highly susceptible to spider mite damage.^[^ ^20^ ^]^ Current research investigating ways to enhance tomato resistance to spider mite infestation primarily focuses on characterizing transcriptional factors that directly regulate JA‐mediated regulatory mechanism that promotes specialized metabolites and trichome formation, including SlMYC1, SlMYB75, SlTHM1, and SlMYB52.^[^ ^4^, ^21^, ^22^, ^23^ ^]^ However, the interplay between JA signaling and metabolic flux, particularly how amino acid metabolism is harnessed to optimize defense, remains underexplored.

Here, we demonstrated that SlAVT6A and SlAVT6B (together referred to as SlAVT6s) are the key genes mediating amino acid export and translocation in vacuole. Overexpression of *SlAVT6A* (*SlAVT6A‐OE*) and knockout of *SlAVT6B* (*SlAVT6B‐ko*) led to an increased density of VI type trichomes, along higher levels of terpenes and gibberellins, which contributed to the tolerance to spider mites. Further analyses revealed that SlWRKY57 directly regulates the expression of *SlAVT6A* and *SlAVT6B*. At the same time, SlJAZ8 interacts with SlWRKY57 and alleviates the effect of SlWRKY57 on SlAVT6s. Our findings highlight the critical role of the SlJAZ8‐SlWRKY57‐SlAVT6A/SlAVT6B module in mediating plant defense against spider mites and provide a novel pathway for investigating how plants modulate primary metabolic flux in response to stress.

## 2 Results

### 2.1 Identification and Functional Validation of SlAVT6A and SlAVT6B

**Figure 1:**
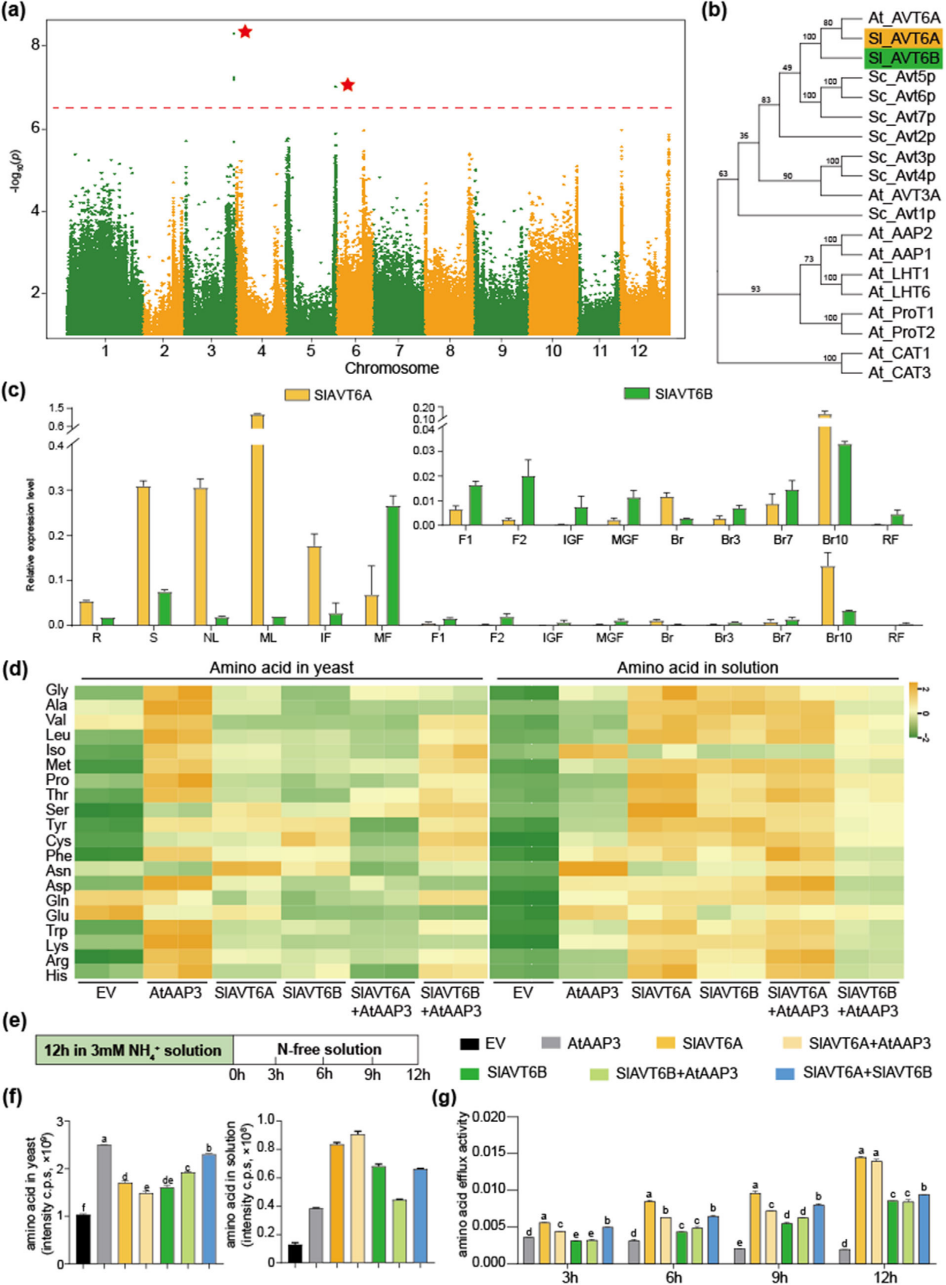
Identification and function validation of SlAVT6A and SlAVT6B. a) Manhattan plot for GWAS of phenylalanine content. Red stars indicate significant QTN on chromosome 3 and 5. b) Phylogenetic analysis of the candidate genes SlAVT6A and SlAVT6B. The maximum‐likelihood method was used to construct the cladogram using MEGA‐X software, with bootstrap values (obtained from 1000 replicates) shown on each node. c) qRT‐PCR results indicate tissue‐specific expression patterns of *SlAVT6A* and *SlAVT6B* in Micro‐Tom. Data are presented as means ± SD, n = 3. d) Heat map of free amino acid concentration in *S. cerevisiae* (left) or external solutions (right) after pre‐treated 3 mm NH_4_^+^ for 12 h. e) Schematic diagram of N treatments. *Saccharomyces cerevisiae* transformants were pre‐treated with 3 mm NH_4_^+^ for 12 h, then exposed to N‐free solution for 0, 3, 6, 9, and 12 h for amino acid concentration determination. f) Amino acid concentration in *S. cerevisiae* (left) or external solutions (right) after 12 h of exposure to N‐free solution. g) amino acid efflux activity after 0, 3, 6, 9, and 12 h of exposure to N‐free solution. Data in (f) and (g) are presented as means ± SD, n = 3, *P*‐values are calculated using the Bonferroni test following ANOVA, significance was defined as *P*≤0.05.

We performed metabolic genome‐wide association study (mGWAS) by integrating the contents of amino acid on tomato accessions.^[^ ^24^ ^]^ The GWAS results indicated that phenylalanine hotspots were mainly located on chromosomes 3 and 5 (**Figure** **1**a). Solyc03g117350, an amino acid transporter, was identified 205 kb downstream of the peak SNP signal 366 703 188 (Figure 1a). Additionally, SNP 562 614 169 was located within the genic region of another amino acid transporters, Solyc05g052300 (Figure 1a). Further study showed that the allele (GG) frequency of Solyc03g117350 increased from 31.25% (PIM) to 81.10% (CER), while the TACTTT allele of Solyc05g052300 rose from 23.08% (PIM) to 96.88% (BIG) (Figure S1, Supporting Information). Phylogenetic analysis revealed that Solyc03g117350 and Solyc05g052300 (i.e., SlAVT6B and SlAVT6A), together with AVT proteins form a monophyletic clade (AVT clade) that also includes AtAVT6A and AtAVT3A (Figure 1b). Consistent with this finding, subcellular localization of SlAVT6A and SlAVT6B showed that fluorescent labeling of GFP fusion proteins coincided with the tonoplast (Figure S2, Supporting Information), similar to other transporters in the AVT family.^[^ ^8^, ^10^ ^]^ Quantitative reverse transcription‐PCR (qRT‐PCR) indicated that SlAVT6A was ubiquitously expressed in most tissues but was expressed at relatively low levels in developing fruits. Expression of SlAVT6B was confined to mature flowers and shoots (Figure 1c).

Putative SlAVT6 orthologs in yeast mediate amino acid bidirectional transport from vacuoles for essential physiological processes.^[^ ^25^ ^]^ To investigate whether SlAVT6s can directly transport amino acids, we employed heterologous expression for SlAVT6A and SlAVT6B with a yeast mutant strain 22Δ10α, which is unable to grow on media containing proteinogenic amino acids or GABA except for Arg as the sole nitrogen (N) source.^[^ ^26^ ^]^ The transformation of SlAVT6A or SlAVT6B did not restore growth on media containing amino acids at concentrations of 2 mm, while transformation of AtAAP3 (the high‐affinity amino acid importer from *Arabidopsis thaliana*) restored growth (Figure S2, Supporting Information).^[^ ^27^ ^]^ We co‐expressed SlAVT6s with AtAAP3 and found that the growth of mutant yeast on amino acid‐containing media was resorted but weaker than AtAAP3 alone (Figure S3, Supporting Information), indicating that SlAVT6A and SlAVT6B may act as amino acid exporters in these strains. To confirm this hypothesis, the amino acid efflux experiment was performed (Figure 1e). In 22Δ10α cells expressing SlAVT6A, SlAVT6B, and co‐expressing SlAVT6A and AtAAP3, the contents of free amino acids were significantly decreased compared those expressing AtAAP3 alone, except for Ser, Tyr, Cys, Asn and Glu (Figure 1d). However, this change was not obvious when SlAVT6B was co‐expressed with AtAAP3 (Figure 1d). We also found that the contents of free amino acid in the liquid culture showed the opposite trend (Figure 1d). We then calculated the total amino acid contents in yeast and liquid culture, the total amino acid concentrations were 32% and 35% lower in 22Δ10α carrying SlAVT6A and SlAVT6B, respectively, compared to AtAAP3 after 12 h of treatment with 3 mm NH_4_^+^ (Figure 1f). The amino acid content in liquid culture was higher in SlAVT6A or SlAVT6B strains compared to yeast expressing AtAAP3 (Figure 1f). The efflux activities of SlAVT6A and SlAVT6B transformants were approximately sevenfold higher than the activity of AtAAP3 after treatment for 12 h in N‐free solution (Figure 1f). These findings indicate that SlAVT6A exhibits a higher efflux activity for amino acid (alanine, valine, leucine, proline, cysteine, phenylalanine, glutamine, tryptophan, lysine, arginine, and histidine) compared to SlAVT6B, which shares substrate specificity but demonstrates markedly reduced transport capacity.

### 2.2 SlAVT6B Interferes with SlAVT6A Activity and Antagonistically Regulates Tomato Growth

**Figure 2:**
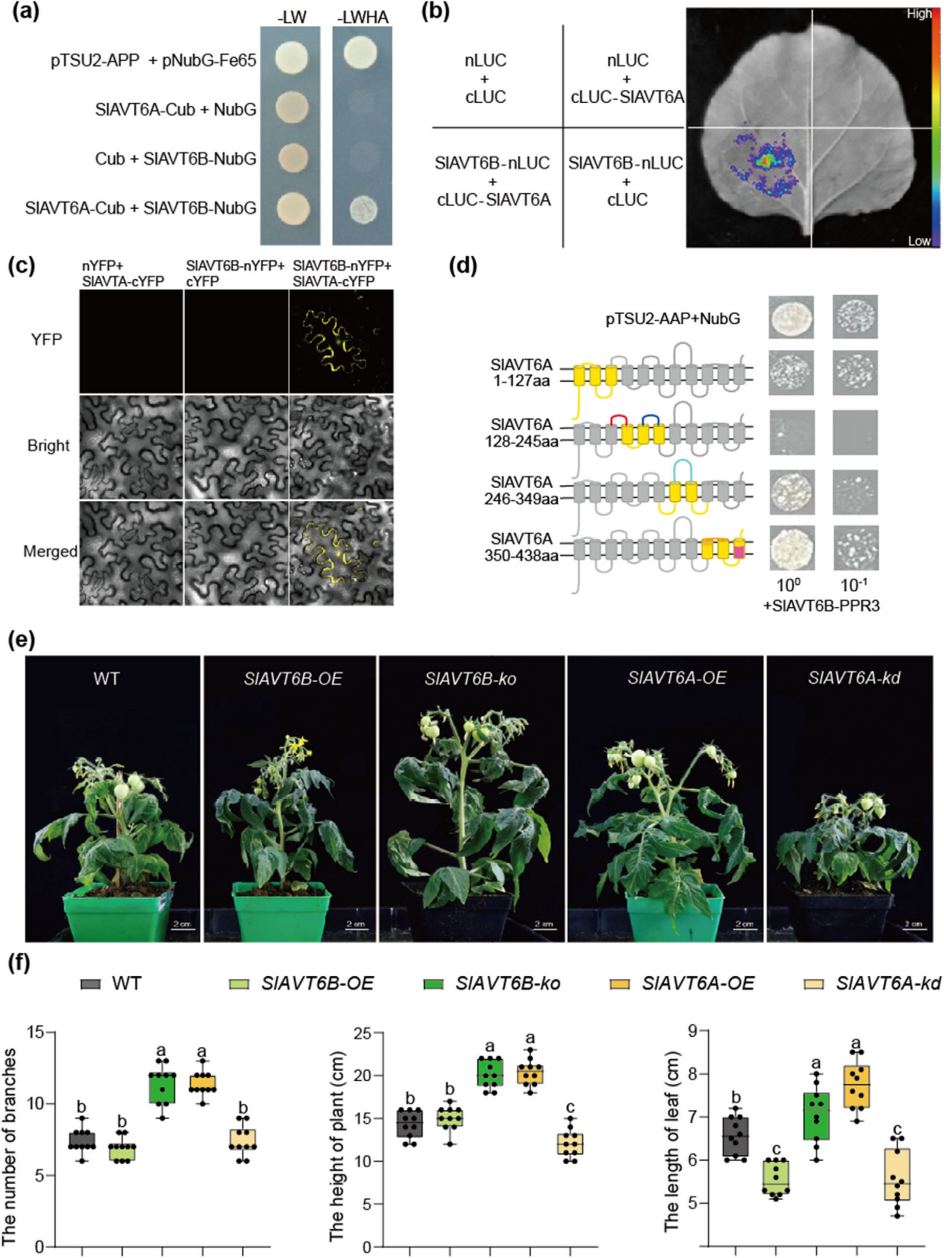
Generation and validation of a structural model predicting that the SlAVT6A‐SlAVT6B heterodimer. a) Interactions between SlAVT6A and SlAVT6B detected by Y2H assay in yeast split‐ubiquitin system. Transformed yeast cells were grown on the control medium, ‐LW (synthetic dropout medium without Leu and Trp), and the selective medium, ‐LWH (synthetic dropout medium without Leu, Trp, His and Ade). b) LCI assay of SlAVT6A with SlAVT6B in tobacco leaves. Coloured scale bars indicate the luminescence intensity in counts per second (cps). c) Bimolecular fluorescence complementation assay analysis. nYFP‐SlAVT6B with cYFP‐ SlAVT6A were co‐transformed into tobacco leaves. nYFP, N‐terminal YFP; cYFP, C‐terminal YFP. nYFP‐SlAVT6B with cYFP, cYFP‐ SlAVT6A with nYFP served as negative controls. Scale bars, 20 µm. d) The interaction of different SlAVT6A truncations and SlAVT6B. In the schematic structures of SlAVT6A (left), gray coloration indicates removed sequences. The right panel indicates interactions between SlAVT6B and different SlAVT6A truncations in the yeast split‐ubiquitin system. e) The phenotype of 2‐month‐old WT, *SlAVT6A* and *SlAVT6B* transgenic plants. Scale bar: 2 cm. f) The number of branches, the height of plant and the length of leaf of WT, *SlAVT6A* and *SlAVT6B* transgenic plants. Data are represented as means ± SD, n = 10, *P*‐values are calculated using Bonferroni test after ANOVA, significance was defined as *P*≤0.05.

When analyzing the efflux capacity of SlAVT6A and SlAVT6B, we found that co‐expression of SlAVT6A and SlAVT6B led to higher amino acids accumulation in yeast, while the amino acid content in the liquid culture significantly decreased to 21.8% of the levels observed with SlAVT6A expressed (Figure 1f). Meanwhile, the efflux activity for amino acids in yeast co‐expressing SlAVT6A and SlAVT6B was significantly lower compared to only SlAVT6A expressing (reduced to 65%), but slightly higher than only SlAVT6B expressing (increased by 1.1‐fold) (Figure 1g). Hence, these results suggest that SlAVT6B affects the transport activity of SlAVT6A. We conducted Y2H assay and the result revealed that SlAVT6A interacts with SlAVT6B (i.e., similar to positive controls; **Figure** **2**a). The luciferase complementation imaging (LCI) assay and bimolecular fluorescence complementation (BiFC) assay further verified that the interaction between SlAVT6A and SlAVT6B proteins preferentially form a heterodimer (Figure 2b,c). To explore the interaction sites between SlAVT6A and SlAVT6B, we performed homology modeling using Phyre2 to predict their structures. Sodium‐coupled neutral amino acid transporter 9 (Protein Data Bank [PDB] ID code 6c08) was selected as template for SlAVT6A and SlAVT6B (Figure S4, Supporting Information).^[^ ^28^ ^]^ The hetero‐dimers of SlAVT6A and SlAVT6B, as predicted by GalaxyHeteromer,^[^ ^29^ ^]^ revealed that the interactions were mediated by insertion of TM10 and TM11 of SlAVT6B into SlAVT6A's outer loop (Figure S5a, Supporting Information). We segmented these residues on the interaction surface of SlAVT6A, and found that residues 1–127, 128–245, and 246–349 did not bind to SlAVT6B, but residues 350–438 did (Figure 2d). The transport pocket of SlAVT6B‐SlAVT6A hetero‐dimer was smaller than that of SlAVT6A alone (Figure S5b, Supporting Information), as analyzed by CASTp.^[^ ^30^ ^]^

To further investigate the function of SlAVT6s in tomato growth, we generated overexpression (OE) transgenic lines for *SlAVT6A* and *SlAVT6B*, and each two independent lines of SlAVT6A (*SlAVT6A‐OE*) and SlAVT6B (*SlAVT6B‐OE*) were selected for further analysis (Figure S6a,b, Supporting Information). Moreover, the knockdown (kd) mutant via RNA interference (RNAi) of SlAVT6A (*SlAVT6A‐kd*) and knockout (ko) mutant of SlAVT6B (*SlAVT6B‐ko*) using CRISPR‐Cas9 technology were generated (Figure S6c–e, Supporting Information). The *SlAVT6A‐OE* and *SlVAT6B‐ko* lines displayed similar phenotypes, with agronomic traits such as branch number, plant height, and internodal length being significantly higher than those of wildtype (Figure 2e,f; Figure S7, Supporting Information). In addition, we found overexpression of SlAVT6B did not affect the growth of tomato, while mutations of *SlAVT6A* repressed plant growth, producing dwarf phenotype and smaller leaves (Figure 2e,f; Figure S7, Supporting Information). Our data suggest that SlAVT6B acts as a regulator affecting the SlAVT6A efflux of amino acid to co‐regulate the growth of tomato.

### 2.3 SlAVT6A and SlAVT6B Exhibit Inverse Effects in Spider Mite Tolerance in Tomato

**Figure 3:**
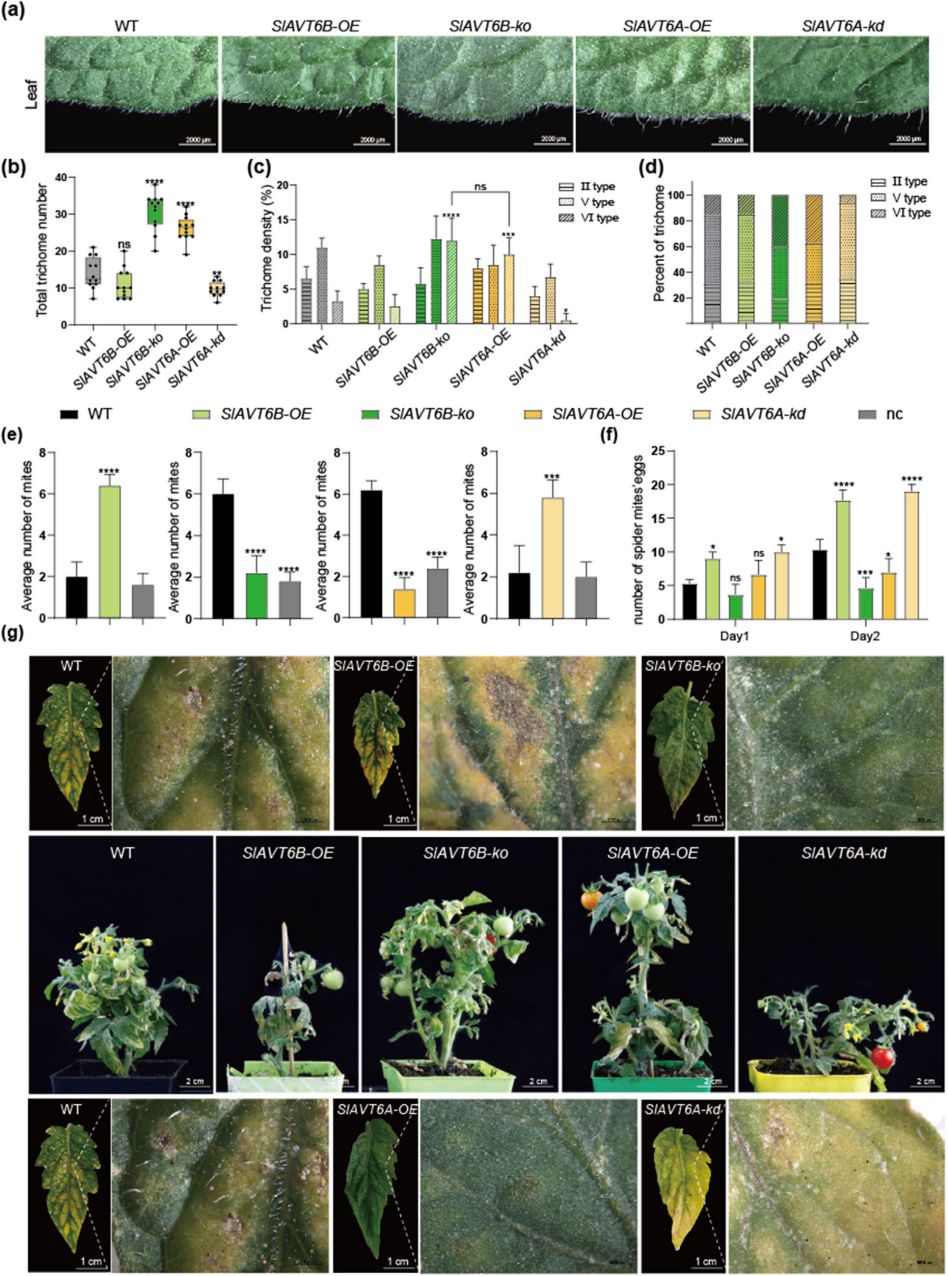
SlAVT6A and SlAVT6B affect trichome density and confer tolerance to tomato. a) Leaf phenotypes of WT, *SlAVT6A‐OE*, *SlAVT6A‐kd*, *SlAVT6B‐OE* and *SlAVT6B‐ko* plants. 6‐week‐old plants were used for all photographs. Scale bar: 2000 µm. b–d) Statistical analysis of trichome abundance (b), density (c) and composition (d) of the leaf in WT, *SlAVT6A*, and *SlAVT6B* transgenic plants. Data are represented as means ± SD, n = 10), *P*‐values are calculated using t‐test, * 0.01<*P*<0.05, ** *P*<0.01. e) Preference experiment to analyze spider mite preference for WT, SlAVT6A, or SlAVT6B transgenic plants. Ten adult female spider mites were positioned in areas equidistant from WT, SlAVT6A, and SlAVT6B leaflets. The number of mites that moved to different leaflets and those that failed to make a choice (nc) were counted 1 h after initiating the assay. Data are presented as means ± SD, n = 10, *P*‐values are calculated using t‐test, * 0.01<*P*<0.05, ** *P*< 0.01, *** *P*<0.001; **** *P*<0.0001. f) Fecundity of spider mites in leaves collected from WT, SlAVT6A and SlAVT6B transgenic plants. Five adult female mites moved to leaf discs (12 mm) of WT and SlAVT6s plants. Eggs were counted at 24 h intervals for 2 days using a microscope. Data are presented as means ± SD, n = 5, *P*‐values are calculated using t‐test, * 0.01<*P*<0.05, ** *P*< 0.01, *** *P*<0.001; **** *P*<0.0001. g) Inoculation of WT, *SlAVT6A* and *SlAVT6B* transgenic plants with spider mites. Fifteen adult female mites were transferred to a single leaf on 45‐day‐old WT, *SlAVT6A* and *SlAVT6B* transgenic plants.

In *SlAVT6A* and *SlAVT6B* transgenic lines, we found *SlAVT6A‐OE* and *SlAVT6B‐ko* showed a high density of trichomes on leaves, stems, and sepals compared with wildtype, while *SlAVT6B‐OE* and the *SlAVT6A‐kd* had fewer trichomes (**Figure** **3**a,b; Figure S8, Supporting Information). To further characterize the role of SlAVT6s in trichome development, we quantified the number of II‐, V‐, and VI‐type trichomes in six‐week‐old wildtype (WT) and transgenic *SlAVT6s* plants.^[^ ^31^ ^]^ Compared with WT, *SlAVT6s* transgenic lines had similar densities of II‐ and V‐type trichomes but a higher number of VI‐type glandular trichomes, which were 3–4 times more abundant in *SlAVT6A‐OE* and *SlAVT6B‐ko* lines than in WT (Figure 3c,d).

Considering that type VI glandular trichomes affect spider mite resistance in tomato, we conducted a two‐choice spider mite feeding assay. During the 2 h feeding, more spider mites preferred leaves from *SlAVT6A‐kd*, *SlAVT6B‐OE* compared to WT, but fewer spider mites preferred *SlAVT6A‐OE* and *SlAVT6B‐ko* to WT (Figure 3e). The number of eggs laid by spider mites is a key factor in host colonization.^[^ ^4^ ^]^ Mites laid more eggs on *SlAVT6A‐kd* and *SlAVT6B‐OE* than on wildtype leaves, while the number of eggs laid on *SlAVT6A‐OE* and *SlAVT6B‐ko* leaves was less than the number laid on wildtype leaves (Figure 3f). To corroborate this result, we also performed a spider mite inoculation assay. After 45 days, *SlAVT6A‐OE* and *SlAVT6B‐ko* lines had smaller lesions and were greener than wildtype plants (Figure 3g). The leaf area affected by chlorotic lesions was consistently 60% the size of lesions on wildtype leaves in *SlAVT6A‐OE* and *SlAVT6B‐ko* plants (Figure S9, Supporting Information). In contrast, lesion size increased by 1.28‐ and 1.35‐fold in *SlAVT6A‐kd* and *SlAVT6B‐OE* lines, respectively (Figure S9, Supporting Information). Consequently, these results are consistent with the growth phenotype, indicating that SlAVT6A and SlAVT6B participate in the growth‐defense response in an antagonistic manner.

### 2.4 SlAVT6s act as Metabolic Valve Reallocating the Amino Acids into Specialized Metabolic Pathways

**Figure 4:**
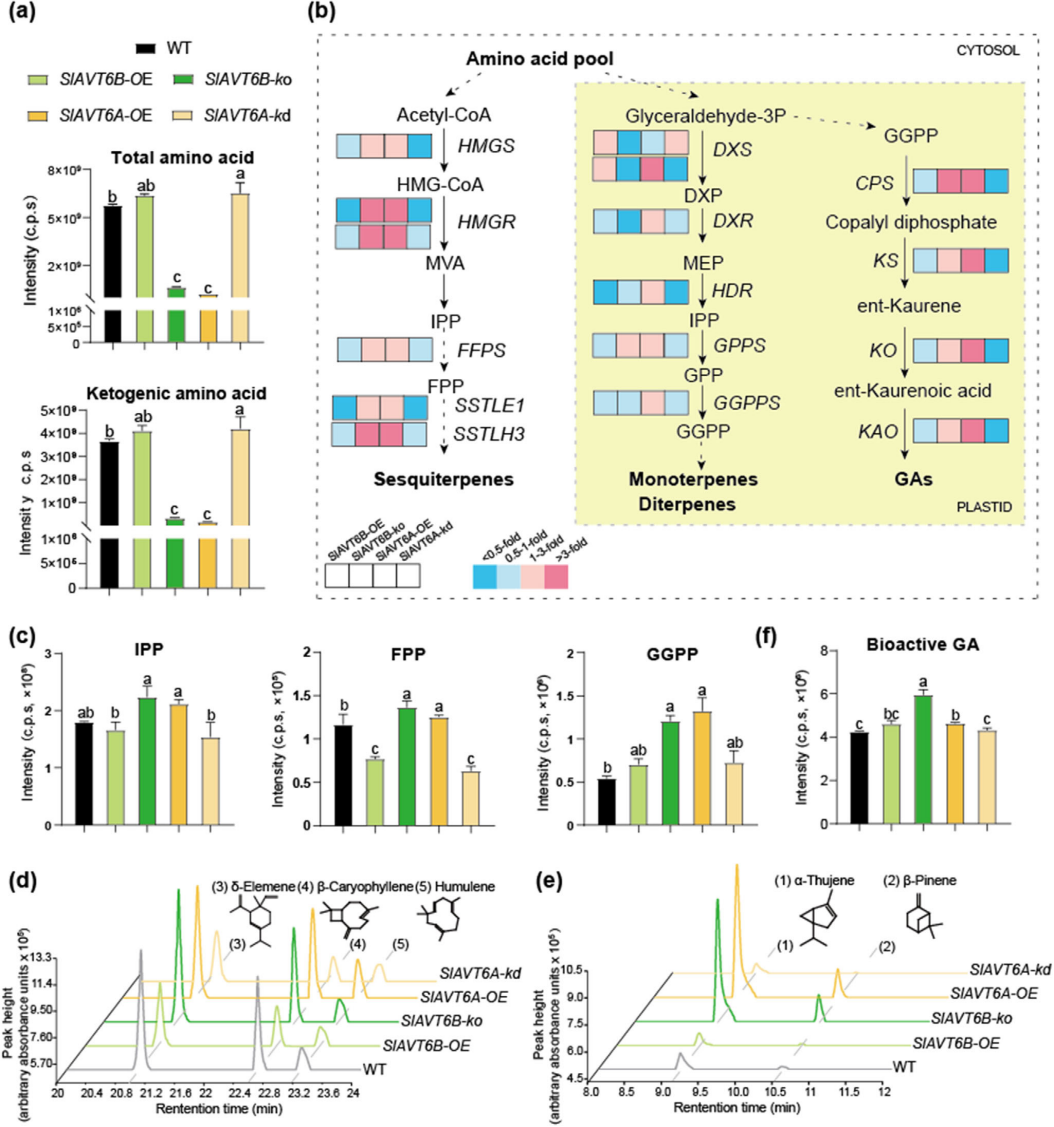
Changes in *SlAVT6A* and *SlAVT6B* expression result in altered primary and secondary metabolic dynamics. a) Total amino acid and ketogenic amino acid levels in WT, *SlAVT6A*, and *SlAVT6B* transgenic plants. Data are presented as means ± SD, n = 3, *P*‐values are calculated using the Bonferroni test following ANOVA, significance was defined as *P*≤0.05. b) Schematic representation of MVA‐, MEP‐, and GA‐metabolic related gene expression in WT, *SlAVT6A*, and *SlAVT6B* transgenic plants. Data are presented as fold change compared to WT. Detailed data are shown in Figure S10 (Supporting Information). Data are presented as means ± SD, n = 3. c) IPP, FPP, and GGPP levels in WT, *SlAVT6A*, and *SlAVT6B* transgenic plants. Data are presented as means ± SD, n = 3, *P*‐values are calculated using the Bonferroni test following ANOVA, significance was defined as *P*≤0.05. d,e) Chromatograms of δ‐Elemene, β‐caryophyllene, humulene, α‐Thujene and β‐Pinene based on GC‐MS analysis from WT, *SlAVT6A*, and *SlAVT6B* transgenic plants leaves. The Y‐axis represents terpene (µg) per gram (g) fresh leaf. f) Levels of bioactive GA in WT, *SlAVT6A*, and *SlAVT6B* transgenic plants. Data are presented as means ± SD, n = 3, *P*‐values are calculated using the Bonferroni test following ANOVA, significance was defined as *P*≤0.05.

To determine whether changes in growth‐defense response in *SlAVT6s* transgenic lines, we first measured leaf amino acid composition and found that total free amino acid levels declined in *SlAVT6A‐OE* and *SlAVT6B‐ko* lines, including glycine, leucine, isoleucine, tyrosine, valine, proline, phenylalanine, tryptophan, methionine, lysine, arginine, and histidine (**Figure** **4**a; Figure S10, Supporting Information). Additionally, we observed that the ketogenic amino acids contents were significantly reduced in *SlAVT6A‐OE* and *SlAVT6B‐ko* lines compared with wildtype (Figure 4a). This suggests that SlAVT6A modulates amino acid availability for specialized metabolic pathways, while SlAVT6B does so by interfering with the transport capacity of SlAVT6A.

Considering the trichome‐phenotype of SlAVT6s transgenic lines, we next investigated the expression of genes related to cytosolic mevalonate (MVA) and plastid methylerythritol phosphate (MEP) pathways in *SlAVT6s* transgenic lines using qRT‐PCR. We found that genes involved in the MVA pathway (*HMGS*, *HMGR1*, *HMGR2* and *FFPS*) and sesquiterpene‐related genes (*SlSSTLE1* and *SlSSTLH3*) were up‐regulated in *SlAVT6A‐OE* and *SlAVT6B‐ko* lines (Figure 4b; Figure S11a, Supporting Information). Moreover, genes related to the MEP pathway (*DXS1*, *DXS2*, *DXR*, *HDR*, *GPPS* and *GGPPS2)* were also induced in *SlAVT6A‐OE* lines, while changes in *SlAVT6B‐ko* lines were inconsistent (Figure 4b; Figure S11b, Supporting Information). Consistent with expression levels of genes, high levels of isopentenyl diphosphate (IPP), farnesyl pyrophosphate (FPP) and geranylgeranyl pyrophosphate (GGPP) accumulated in *SlAVT6A‐OE* (≈1.2, 1.1, and 2.5‐fold higher, respectively) and *SlAVT6B‐ko* (≈1.2, 1.2, and 2.2‐fold higher, respectively) lines (Figure 4c). Additionally, we quantified the volatile terpenes in leaves collected from WT and *SlAVT6s* transgenic plants. Two monoterpenes (α‐thujene and β‐pinene), six sesquiterpenes (δ‐elemene, β‐elemene, caryophyllene, alloaromadendrene, humulene, and germacrene D) and one diterpene (neophytadiene) were identified. The terpene quantification revealed that total terpene levels were higher in *SlAVT6A‐OE* and *SlAVT6B‐ko* lines (4.32‐, 1.86‐fold, respectively), while α‐thujene, β‐pinene, δ‐elemene, caryophyllene, and humulene levels were 13.2‐, 15.8‐, 3.7‐, 1.9‐, and 1.8‐fold higher using gas chromatography‐mass spectrometry (GC‐MS), respectively, compared to wildtype (Figure 4d,e). *SlAVT6A‐kd* and *SlAVT6B‐OE* lines produced fewer terpenes than wildtype, and levels of caryophyllene and humulene were significantly lower (Figure 4d). Together, these findings suggest that SlAVT6s reprogram secondary metabolism by adjusting amino acid allocation, contributing to the volatile terpenes involved in insect resistance.

Gibberellin is involved in type VI glandular trichome development,^[^ ^32^, ^33^ ^]^ and concentrations of GGPP, the precursor of gibberellin, increased in *SlAVT6A‐OE* and *SlAVT6B‐ko* plants. We examined expression levels of genes related to gibberellin biosynthesis and gibberellin‐dependent trichome regulators to investigate whether SlAVT6s affect gibberellin content, thereby regulating type VI glandular trichome development. Transcript levels of gibberellin biosynthesis genes, such as *ent*‐copalyl diphosphate synthase (*CPS*), *ent*‐kaurene synthase (*KS*), *ent*‐kaurene oxidase (*KO*), and *ent*‐kaurenoic acid oxidase (*KAO*),^[^ ^34^ ^]^ were up‐regulated in *SlAVT6A‐OE* and *SlAVT6B‐ko*, but silencing *SlAVT6A* and overexpressing *SlAVT6B* prevented upregulation (Figure 4b; Figure S12, Supporting Information). Furthermore, bioactive gibberellin increased in *SlAVT6A‐OE* and *SlAVT6B‐ko* plants (Figure 4f). Additionally, leaves of *SlAVT6A‐OE* and *SlAVT6B‐ko* that were treated with paclobutrazol (PAC) had a phenotype similar to that of wildtype plants (Figure S13a, Supporting Information), and the number of VI trichomes was similar to the number of trichomes in wildtype (Figure S13b,c, Supporting Information). These results suggest that SlAVT6s may regulate trichome development by altering levels of amino acids related to gibberellin biosynthesis.

### 2.5 WRKY57 Regulates the Coordination of SlAVT6s in Tomato

**Figure 5:**
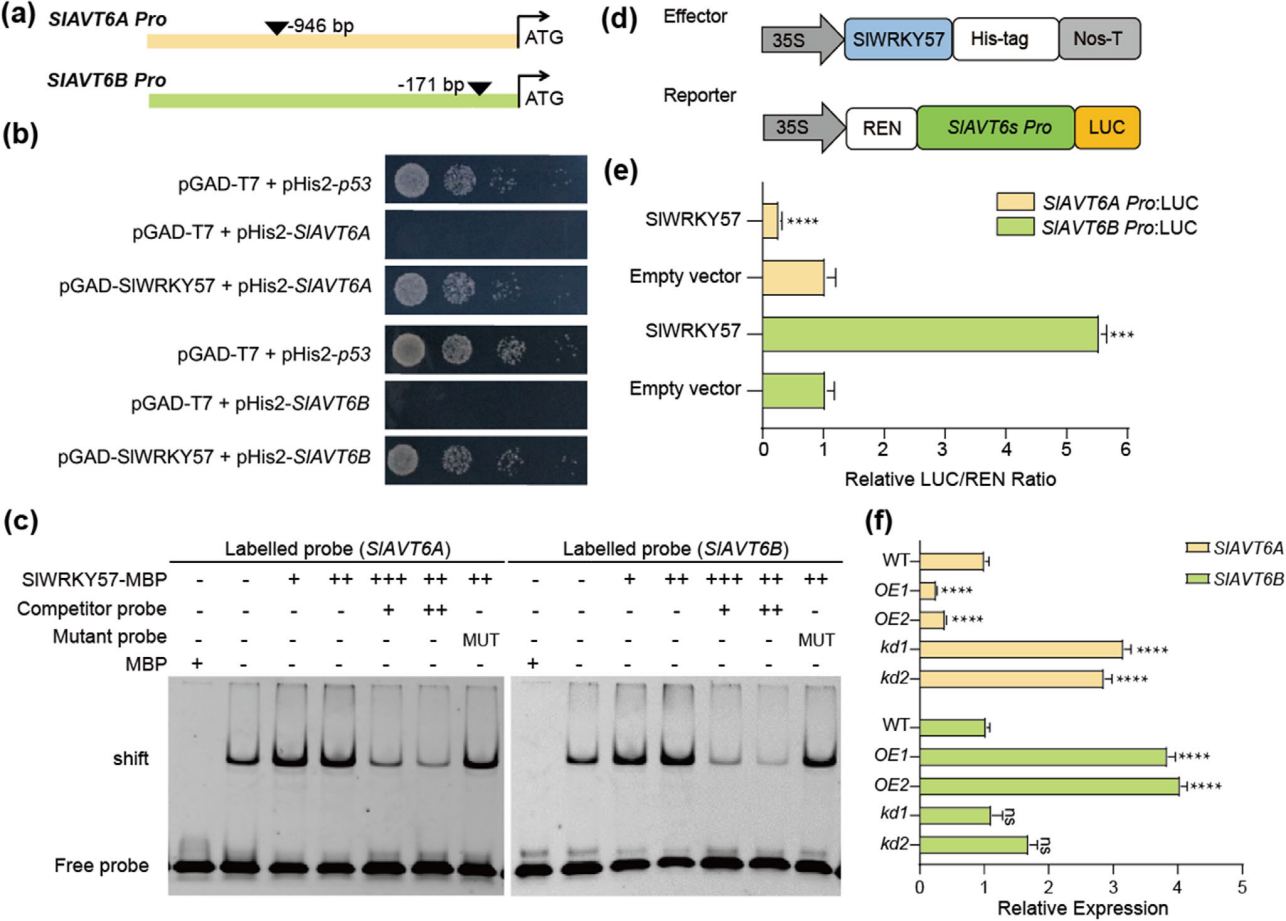
WRKY57 regulates the coordination of SlAVT6A and SlAVT6B in tomato. a) Schematic diagram of the SlAVT6A and SlAVT6B promoter. The location of the W‐box is indicated by black inverted triangles. b) Yeast one‐hybrid indicates that SlWRKY57 interacts directly with the W‐box in the promoter of *SlAVT6A* and *SlAVT6B*. Transformed yeast cells were grown on control medium, ‐LW (synthetic dropout medium without Leu or Trp), and the selective medium, ‐LWH (synthetic dropout medium without Leu, Trp, or His). c) Electrophoretic mobility shift assays (EMSA) of SlWRKY57 binding to the promoter of *SlAVT6A* and *SlAVT6B*. The SlWRKY57 protein‐DNA complexes were separated on 3% native polyacrylamide gels. “−” and “+” represent absence and presence, respectively. “++” and “+++” indicate increasing amounts purified protein. “MUT” represents mutation probes that were used for competition. d) Schematic diagrams of the effector and reporter constructs used in dual‐luciferase (dual‐LUC) assays. Effector constructs contain the SlWRKY57 coding sequence driven by the 35S promoter. *SlAVT6s pro*:LUC were used as reporter constructs. REN expression under the 35S promoter was used as internal control. The His driven by 35S promoter was used as control. e) Dual‐LUC assay in *N. benthamiana* leaves using the constructs shown in (d). LUC/REN control ratios were set as 1. Data are presented as means ± SD, n = 3, *P*‐values are calculated using t‐test, *** *P*<0.001. f) Expression levels of *SlAVT6A* and *SlAVT6B* in *SlWRKY57* transgenic plants. Data are presented as means ± SD of two replicates from two *SlWRKY57‐OE* and two *SlWRKY57‐kd* independent lines. *P*‐values are calculated using one‐way ANOVA: * 0.01<*P*<0.05, ** *P*<0.01, *** *P*<0.001, **** *P*<0.0001.

To further investigate the function of SlAVT6s, we analyzed their promoters using PlantRegMap (http://plantregmap.gao‐lab.org/binding_site_prediction.php) and performed co‐expression network analysis and identified a regulator SlWRKY57. The w‐box is a potential binding site for SlWRKY57 and was detected in the promoter of *SlAVT6s* genes (**Figure** **5**a). The regulation of SlWRKY57 on *SlAVT6s* was confirmed in vitro (using Y1H and EMSA assays) and in vivo (using the dual‐LUC assay). The result of the Y1H assay revealed that SlWRKY57 binds directly with the *SlAVT6s* promoter (Figure 5b). EMSA assays further validated the binding affinity of SlWRKY57 to *SlAVT6s* promoters (Figure 5c). A dual‐luciferase assay confirmed that *SlAVT6s* promoter activity is regulated by SlWRKY57. Transcriptional activity of the *SlAVT6A* promoter was suppressed, whereas the transcriptional activity of *SlAVT6B* was activated by SlWRKY57 relative to control (Figure 5d,e). To further characterize how SlWRKY57 regulates *SlAVT6s*, the transgenic plants of SlWRKY57 were generated (Figure S14, Supporting Information). The results of qRT‐PCR analysis indicated that expression levels of *SlAVT6A* were significantly lower in *SlWRKY57‐OE* lines but were significantly higher in *SlAVT6B* compared to wildtype (Figure 5f). These findings suggest that SlWRKY57 targets SlAVT6s directly to regulate their transcription.

### 2.6 The SlWRKY57‐SlAVT6s Module Participates in JA‐Mediated the Tolerance of Spider Mite

**Figure 6:**
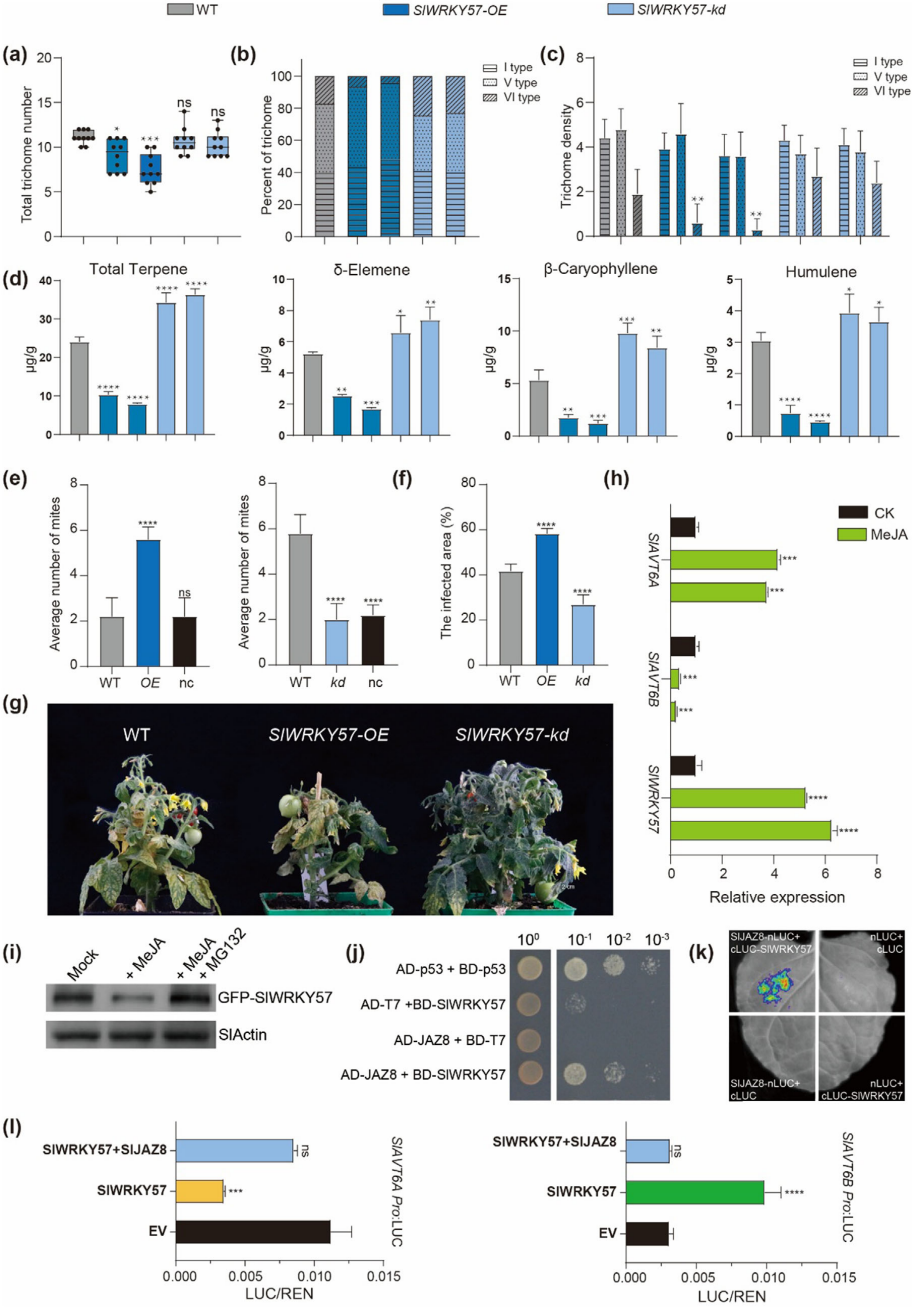
The SlWRKY57‐SlAVT6A/SlAVT6B module is involved in JA‐mediated tolerance of spider mites. (a‐c) Statistical analysis of trichome abundance (a), density (b) and composition (c) of leaves in WT and SlWRKY57 transgenic plants. Data are presented as means ± SD, n = 10, *P*‐values are calculated using t‐test: * 0.01<*P*<0.05, ** *P*<0.01, *** *P*<0.001. (d) Quantification of terpenes in WT and *SlWRKY57* plants based on GC‐MS analysis. Data are presented as means ± SD of three replicates from two SlWRKY57 independent lines. *P*‐values are calculated using one‐way ANOVA: * 0.01<*P*<0.05, ** *P*<0.01, *** *P*<0.001, **** *P*<0.0001. e) Spider mite preference experiment for WT and two *SlWRKY57* independent lines. Ten adult female spider mites were positioned areas equidistant from WT and *SlWRKY57* leaflets. The number of mites that moved to different leaflets and those that failed to make a choice (nc) were counted 1 h after assay initiation. Data are presented as means ± SD, n = 10, *P*‐values are calculated using t‐test: * 0.01<*P*<0.05, ** *P*< 0.01, *** *P*<0.001, **** *P*<0.0001. f) Infected area in WT and two *SlWRKY57* independent lines 45 days after inoculation with spider mites. Data are presented as means ± SD, n = 5, *P*‐values are calculated using t‐test: * 0.01<*P*<0.05, ** *P*< 0.01, *** *P*<0.001, **** *P*<0.0001. g) Inoculation of WT and *SlWRKY57* plants with spider mites. Fifteen adult female mites were transferred to a single leaf on 15‐day‐old WT and *SlWRKY57* plants. h) Expression levels of *SlWRKY57*, *SlAVT6A*, and *SlAVT6B* after MeJA treatment. Data are presented as means ± SD, n = 3, *P*‐values are calculated using two‐way ANOVA: * 0.01<*P* <0.05, ** *P*<0.01, *** *P*<0.001, **** *P*<0.0001. i) Immunoblot analysis of the effects of MeJA on protein levels of SlWRKY57‐GFP. SlWRKY57 in total protein extracts from *N. benthamiana* leaves expressing SlWRKY57‐GFP with and without MeJA treatment and/or 20 µM MG132. SlWRKY57 protein levels as determined by immunoblot using anti‐GFP antibodies. Actin was used as a loading control. j) Yeast two‐hybrid analysis of SlWRKY57 and SlJAZ8 interactions. Transformed yeast cells were grown on the medium SD/‐Trp/‐Leu and selective media SD/‐Trp/‐Leu/‐His/‐Ade. k) Split luciferase complementation assay demonstrated the in vivo interaction between SlWRKY57 and SlJAZ8. Three sets of construct combinations, including nLUC and cLUC, SlJAZ8‐nLUC and cLUC, nLUC, and SlWRKY57‐cLUC, were transformed and used as negative controls. l) SlJAZ8 undermines SlWRKY57's regulation on *SlAVT6A* and *SlAVT6B* gene expression. Three independent transfection experiments were performed. Data are presented as means ± SD, n = 3, *P*‐values are calculated using t‐test: *** *P*<0.001; **** *P*<0.0001.

The interaction between SlWRKY57 and the *SlAVT6s* promoter confers spider mite tolerance by affecting trichome development and terpene content. Thus, we also investigated the phenotype and related gene expression of *SlWRKY57* transgenic lines. Compared with wild type, trichome density in *SlWRKY57‐OE* lines decreased 18% to 32%, mainly in VI‐type trichomes (Figure S15, Supporting Information; **Figure** **6a–c**). Furthermore, the levels of monoterpene and sesquiterpene were reduced to 50% compared to the wild type in *SlWRKY57‐OE* plants (Figure 6d; Figure S16, Supporting Information). Next, we performed a two‐choice spider mite feeding assay to examine whether SlWRKY57 affects insect resistance in tomato. During the two‐hour feeding, spider mites preferred leaves from *SlWRKY57‐OE* to wildtype leaves, and the number of mites observed in *SlWRKY57‐kd* lines was significantly lower than the number observed in wildtype plants (Figure 6e). To provide further verification, we performed a mite feeding assay. 6‐week‐old transgenic and wildtypes were infested with adult mites. After 45 days, severe damage was observed in *SlWRKY57‐OE* and wildtype plants (Figure 6g; Figure S17, Supporting Information). By contrast, the inoculated leaves of *SlWRKY57‐kd* lines remained green and had relatively few signs of macroscopic damage (Figure 6g; Figure S17, Supporting Information). Quantitation of the number of damaged leaves and leaf area occupied by lesions demonstrated that wildtype and *SlWRKY57‐OE* leaves suffered significantly more damage than those of *SlWRKY57‐kd* plants (Figure 6f). These results confirm that SlWRKY57 regulates *SlAVT6s* expression to help control amino acid allocation for VI‐type trichome development and terpene biosynthesis.

Previous work has shown that AtWRKY57, a component of the JA‐mediated signaling pathway, regulates the resistance of Arabidopsis (*Arabidopsis thaliana*) to *B. cinerea*.^[^ ^35^, ^36^ ^]^ To investigate the role of the SlWRKY57‐*SlAVT6s* module in JA‐mediated resistance, we treated wildtype plants with MeJA and measured expression levels of *SlWRKY57* and *SlAVT6s*. This treatment resulted in increased expression of *SlWRKY57* and *SlAVT6A*, while the transcript level of *SlAVT6B* was lower following treatment (Figure 6h). Consistent with previous work,^[^ ^35^ ^]^ we found that MeJA treatment resulted in the degradation of the SlWRKY57 protein (Figure 6i), which affects downstream SlAVT6s expression levels. Consistent with other researches, our Y2H and LCI assay revealed that SlWRKY57 interacts with SlJAZ8 (Figure 6j,k). To validate the hypothesis that JAZ may interfere with the interaction between SlWRKY57 and SlAVT6s, dual‐LUC analysis was performed. The results indicate that the transcript responsiveness of the *SlAVT6s* promoter with SlWRKY57 is weakened in the presence of SlJAZ8 (Figure 6l), suggesting that the SlWRKY57‐SlAVT6A‐SlAVT6B module plays a role in JA‐mediated spider mite tolerance.

## 3 Discussion

**Figure 7:**
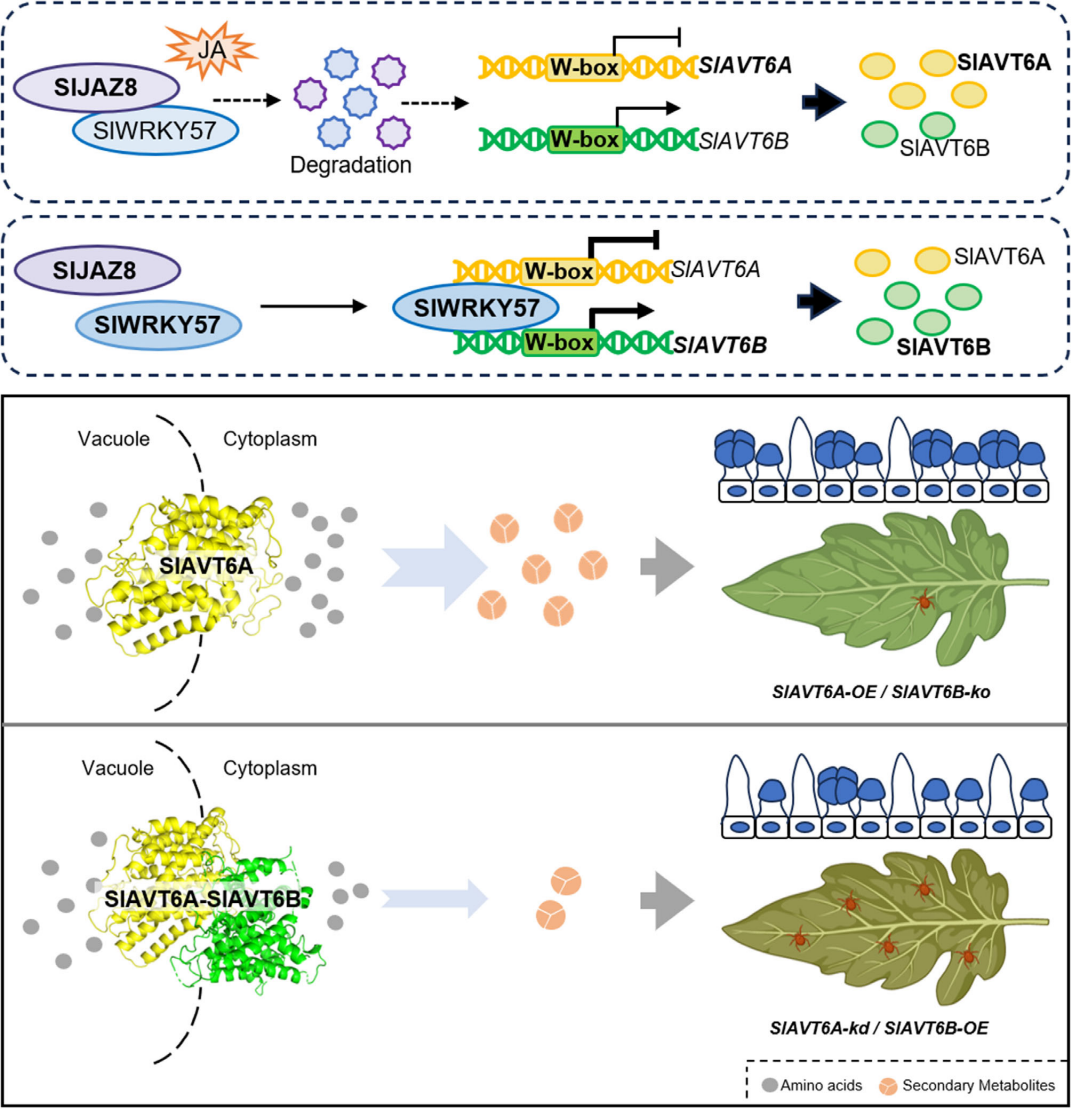
Proposed model of the SlWRKY57‐SlAVT6A/SlAVT6B module's function in enhancing spider mite resistance by modulating intracellular amino acid homeostasis in tomato. SlJAZ8 interacts with SlWRKY57 and promotes its degradation upon JA treatment, which directly suppresses SlAVT6A/SlAVT6B expression to adjust the intracellular amino acid homeostasis. The efflux activity of SlAVT6A is much greater than that of SlAVT6B, and SlAVT6B inhibits the efflux activity of SlAVT6A. The overexpression of *SlAVT6A* or knockout of *SlAVT6B* in tomato induces the accumulation of the amino acids associated with terpene synthesis and increased trichome density, thus increasing spider mite tolerance. The figure was created using BioRender.com.

Plants precisely adjust primary metabolism by altering gene expression and metabolic pathways to provide necessary energy and precursor molecules, thereby increasing the synthesis of defensive secondary metabolites and enhancing their adaptation and resistance capabilities.^[^ ^5^ ^]^ However, the mechanisms underlying the balance between primary metabolic flux and secondary metabolism require further elucidation. In this study, we found that the vacuolar amino acid transporters SlAVT6A and SlAVT6B play distinct roles, with the former acting as the primary amino acid efflux protein, while the latter, in addition to having weaker efflux activity, primarily interferes with the amino acid efflux activity of SlAVT6A. SlAVT6A and SlAVT6B regulate amino acid flux to terpene and gibberellin metabolism, which participate in plant defense and growth response. This study showed that MeJA treatment induced SlWRKY57 protein degradation and consequently increased SlAVT6A and suppressed SlAVT6B, mediated by an JA signaling regulator SlJAZ8. Furthermore, SlWRKY57 is a negative regulator to the resistance of spider mites, *Botrytis cinerea* and salt tolerance.^[^ ^35^, ^37^ ^]^ These findings identified SlWRKY57‐SlAVT6A/SlAVT6B functions as a key coordinating module in the regulation of plant growth and stress responses by modulating amino acid homeostasis (**Figure** **7**), providing a potential for breeding programs to improve spider mite tolerance in tomatoes by altering metabolite fluxes.

### 3.1 SlAVT6B Tune the Amino Acid Export Capacity of SlAVT6A

In plants, amino acid transporters are involved in various physiological processes, affecting growth and development, yield and quality, as well as stress responses. Such as, AtAAP5 and OsLHT1 have been identified as contributors to root absorption of amino acids, which target neutral and acidic aminos acids.^[^ ^38^, ^39^ ^]^ Later, AtAAP3, AtAAP8, and AtUMAMIT18, are important for phloem loading and are thus crucial to grain yields.^[^ ^40^, ^41^ ^]^ In addition, GmProT1 and GmProT2 can improve salt and drought tolerance,^[^ ^42^ ^]^ and the overexpression of AtLHT1 can facilitate the uptake of R‐beta‐homoserine (RBH) and beta‐aminobutyric acid (BABA) to prime disease resistance.^[^ ^4^ ^]^ Here, we characterized the transport capabilities of SlAVT6A and SlAVT6B, revealing their capacity to drive the efflux of amino acids in heterologous yeast (Figure 2). Transport experiments in yeast mutant strains demonstrated that SlAVT6A has stronger efflux activity than SlAVT6B. While SlAVT6B has a broad, low expression pattern, SlAVT6A is highly expressed in vegetative organs where amino acids are actively being transferred and used. Thus, we propose that SlAVT6A acts as a generalist transporter in different tissues to transport amino acids, which enriches the function of amino acid transporters.

Our study verified that co‐expression of SlAVT6A and SlAVT6B accumulated fewer amino acids than SlAVT6A alone, and the phenotype of SlAVT6B mutation was consistent with that of SlAVT6A overexpression lines. Therefore, SlAVT6A and SlAVT6B might interact for amino acid transport in vacuole. We provided genetic and biochemical evidence for SlAVT6B can dimerize with SlAVT6A and affect the transport activity of SlAVT6A. Previous studies had identified transporters can form specific dimer pairings, which affect its transport activity by adjusting the binding ability of substrate and the structure of transmembrane, such as ATP binding cassette (ABC) transporters, sulfate (SULTR) transporters, PIN‐FORMED (PIN) protein.^[^ ^43^, ^44^, ^45^ ^]^ However, these dimer combinations have not been studied in amino acid transporters in plants. In our study, SlAVT6A plays a major role in mediating amino acid transport from vacuoles to the cytoplasm and in remobilizing amino acids stored in vacuoles. SlAVT6B, acting as a regulator of SlAVT6A, pairs with SlAVT6A in various tomato tissues to regulate its transport activity, thereby affecting amino acid homeostasis in the cytoplasm. These findings provide a model for how amino acid transporters regulate amino acid homeostasis in plant cells. Further research is needed to identify other factors that contribute to interactions between SlAVT6A and SlAVT6B and how these interactions regulate amino acid efflux to vacuoles.

### 3.2 SlAVT6s Remobilize Amino Acid Flux to Secondary Metabolism Confers Spider Mite Tolerance

When plants synthesize more amino acids than immediately needed, these excess amino acids are typically transported to vacuoles for storage, to be reused when necessary. This storage mechanism helps maintain cellular amino acid balance and provides essential resources during environmental stress or nutrient deficiencies in plants.^[^ ^46^ ^]^ The differences in amino acid permease activities between SlAVT6A and SlAVT6B may lead to the differences in transport efficiencies for amino acids, contributing to the differences in free amino acid content and phenotype of *SlAVT6A* and *SlAVT6B* transgenic lines. Notably, *SlAVT6A‐OE* and *SlAVT6B‐ko* leaves showed a decrease in twelve amino acids, including leucine, lysine, tryptophan, phenylalanine, and tyrosine. As organic nitrogen sources, we found that the nitrogen assimilation pathways were upregulated in *SlAVT6A‐OE* and *SlAVT6B‐ko* plants relative to wildtype (Figure S18, Supporting Information), which exhibited a better growth trend.

Spider mite tolerance is crucial for normal growth, yield, and quality in tomato.^[^ ^47^ ^]^ Trichome density and terpene content are important mechanical and chemical barriers against insects, respectively.^[^ ^48^ ^]^ Notably, emerging evidence suggests terpenes exhibit ecological multifunctionality. Certain sesquiterpenes such as β‐caryophyllene not only deter herbivores but also serve as attractants for natural enemies of pests, exemplified by their role in recruiting predatory mites *Phytoseiulus persimilis*.**^[^** ^49^ **^,^** ^50^ **^]^** This dual functionality implies terpene metabolism may orchestrate a dynamic defense network balancing direct and indirect protective strategies. While some tolerance genes have been identified, most are transcription factors.^[^ ^14^, ^21^, ^23^ ^]^ Ketogenic amino acids can act as primary precursors to terpene metabolism through MVA and MEP pathways.^[^ ^2^, ^51^ ^]^ We also observed that genes and intermediate metabolites of the MVA and MEP pathways are upregulated, leading to an increase in terpene and gibberellin in plants from *SlAVT6A‐OE* and *SlAVT6B‐ko* lines. These metabolic changes were correlated with enhanced spider mite resistance through higher terpene contents, accumulated gibberellin and increased density of glandular trichome. Furthermore, yields from *SlAVT6A‐OE* and *SlAVT6B‐ko* lines were higher than wildtype following spider mite inoculation (Figure S19, Supporting Information). We propose that SlAVT6A and SlAVT6B regulate the flux of amino acid to maintain a stable equilibrium that precisely controls the contents of the specialized metabolite and that this equilibrium can shift in different environments.

### 3.3 SlWRKY57 Participates in JA Signaling by Interacting with SlJAZ8 and Coordinately Regulates Downstream SlAVT6A and SlAVT6B

Processes maintaining amino acid homeostasis are influenced by environmental signals, yet the mechanism by which transcription factors regulate amino acid transporters in plants is not well understood. In our study, extensive experiments demonstrated that the SlWRKY57 directly inhibits the transcription of *SlAVT6A* and induce the transcription of *SlAVT6B*. Further investigation revealed that SlWRKY57 overexpression led to amino acid accumulation, accompanied by lower terpene content and trichome density. These results suggest that the overexpression of SlWRKY57 can be attributed to a defect in amino acid transport, wherein SlAVT6A transporter activity is inhibited and the upregulated expression of SlAVT6B further suppresses SlAVT6A transporter activity, ultimately increases susceptibility to spider mites.

The Arabidopsis homolog of SlWRKY57, AtWRKY57, is a negative regulator against *B. cinerea* through the JA signaling pathway.^[^ ^35^ ^]^ JA plays a crucial role in trichome development, terpene accumulation, and spider mite resistance in various plant species.^[^ ^15^, ^16^, ^18^, ^52^ ^]^ Our findings suggest that resistance to spider mites conferred by the SlWRKY57‐SlAVT6A/SlAVT6B module is related to the JA pathway. The protein levels of SlWRKY57 decreased significantly under MeJA treatment. Additionally, SlAVT6A expression increased with MeJA treatment, while SlAVT6B expression decreased. We also demonstrated that SlWRKY57 physically interacts with SlJAZ8 and that their co‐expression alleviates changes in the expression of reporter genes *ProSlAVT6A:LUC* and *ProSlAVT6B:LUC*. Our results suggest that, similar to its function in Arabidopsis, SlWRKY57 affects spider mite resistance through JA signaling and competition with JAZ8 to regulate downstream target genes SlAVT6A and SlAVT6B.^[^ ^36^ ^]^ Thus, SlJAZ8, SlWRKY57, SlAVT6A and SlAVT6B form a complex regulatory module characterized by reciprocal interactions at multiple levels, including transcriptional control, modulation of transactivation activity, and regulation of protein stability.

In this study, we show that the flux from amino acids to terpene and gibberellin leads to tolerance to spider mites in tomato. The expression of SlAVT6A and SlAVT6B is involved in the reallocation of amino acids in vacuoles, which is associated with terpenes accumulation, GA synthesis and trichome development, contributing to spider mite tolerance in tomato. Meanwhile, our work shows that SlJAZ8‐SlWRKY57‐SlAVT6A/SlAVT6B module is involved in JA signaling mediated spider mite resistance by redistributing amino acids stored in vacuoles to secondary metabolic pathways (Figure 7), revealing a new strategy for improving plant resistance and yield in future crop breeding by modulating primary metabolic flux to secondary metabolites.

## 4 Experimental Section

### Plant Material Cultivation

Tomato (*Solanum lycopersicum*) cv MicroTom were used as WT in this study with seeds purchased from PanAmerican Seed. Seedlings were grown greenhouse under a 16 h light and 8 h dark at 24 °C. *Nicotiana benthamiana* was grown in a greenhouse with the conditions described above for subcellular localization and dual‐luciferase reporter assay. For spatial and temporal expression analysis, tissues were harvested according as described previously.^[^ ^53^ ^]^ Fruit yield was measured as previously described.^[^ ^54^ ^]^

For PAC (Paclobutrazol) treatment, 4‐week‐old tomato plants were sprayed with 100 µm PAC (Sigma–Aldrich) in sterile water; the control plants were sprayed with sterile water. Newly developed stems and leaflets were collected for trichome morphology analysis, RNA extraction and volatile terpene measurements 1 month later.

### Tomato Accessions and Genome‐Wide Association Study (GWAS)

A total of 1 860 736 single nucleotide polymorphisms (SNPs; minor allele frequency >5% and missing rate <10%) for 236 accessions (Table S1, Supporting Information) were used to perform the genome‐wide association analysis, which included 20 PIM accessions, 77 CER accessions, and 139 BIG accessions. Efficient Mixed‐Model Association eXpedited (EMMAX) was used to conduct all associations.^[^ ^55^ ^]^ GWAS analysis was conducted as described previously.^[^ ^24^ ^]^ The genome‐wide significance threshold (2.97×10^−7^) of the gene‐based GWAS were determined following Bonferroni correction.^[^ ^56^ ^]^

### Constructs and Generation of Transgenic Plants

For the overexpression of *SlAVT6A* and *SlAVT6B*, the full‐length coding sequence of *SlAVT6A* and *SlAVT6B* was amplified using KOD Fx (TOYOBO) and cloned into the pDONR207 vector and further transfer of the resulting plasmid to the pBI121 vector using Gateway Clonase II enzyme mix (Invitrogen). To create mutants of *SlAVT6A*, the 300 bp target sequence of *SlAVT6A* was designed on the website (https://vigs.solgenomics.net/) and cloned into pK7GWIWG2R (II) vector using Gateway Clonase II enzyme mix (Invitrogen). To produce mutations in the coding sequence of *SlAVT6B*, the recombinant pTX41 vector was designed according to the previous report.^[^ ^57^ ^]^ Two target sites were designed in the website (http://crispr.hzau.edu.cn/cgi‐bin/CRISPR2/CRISPR) and cloned into the pTX41 vector using Golden Gate (Vazyme Biotech C112‐01/02). The plasmid with the correct insertion was introduced into *Agrobacterium tumefaciens* strain EHA105 and tomato transformation was done as described previously.^[^ ^58^ ^]^ All primers used in this experiment were listed in the Table S2 (Supporting Information).

### Function Study of SlAVT6A and SlAVT6B in Yeast System

The full‐length cDNA of *SlAVT6A* and *SlAVT6B* were transferred from pDONR207 vector to destination ADH1 vector and transformed into wild type 2234c yeast and 22Δ10α mutant yeast alone or together with AtAAP3, respectively. Transformants were grown in SD/‐Ura liquid medium and spotted onto solid medium containing either 2 mm amino acid as sole nitrogen source or 1 mm ammonium sulfate in an incubator maintained at 30 °C for 2–3 days. For amino acid accumulation test, yeasts were grown in liquid SD/‐Ura medium to an OD600 of 1, and collected and washed with distilled water three times. Then, yeast samples were transferred to the new liquid medium for 24 h with 3 mm NH_4_^+^ as sole source of nitrogen, followed by to determine amino acid concentration. For the amino acid efflux activity test, yeasts were grown in a minimal medium to an OD600 of 1 and treated with 3 mm NH_4_^+^ for 12 h. Yeast samples were collected and washed three times, and then transferred into the N‐free medium for 0, 2, 4, 6, and 8 h. Yeast samples and external solution were collected to determine amino acid concentration. The amino acid efflux activity was calculated as the amount of amino acid in external solution divided by the amount of amino acid in yeast and external solution. At least three biological replicates were included for each assay with three technical replicates for each biological replicate.

### Analysis of Trichome Morphology and Density

The trichomes were visualized either on the stereomicroscope (DFC550 LEICA). The density of trichomes on the adaxial surface of leaves was determined by counting trichomes on the third leaf in each 6‐week‐old tomato plant. For the analysis of trichome density on stems, the stems were chosen from the third internodes of 6‐week‐old plants. For each line, about 6–7 images were taken for the analysis.

### Total RNA Extraction and Quantitative Real‐Time PCR

Total RNA was extracted from tomato leaves by TransZol Up reagent (TransGen Biotech). First strand cDNAs were synthesized using the Evo M‐MLV RT kit (AG11706; Accurate Biotechnology, Beijing, China). qPCR was performed using the one‐step gDNA removal and cDNA synthesis supermix (TransGen Biotech). The qRT‐PCR program was performed with 2×Q3 SYBR qPCR Master mix (Universal) (TOLOBIO). Relative expression was calculated using the 2^−ΔΔ^CT method, and Slactin were used as reference genes.^[^ ^59^ ^]^ The primers used for qRT‐PCR reactions are listed in Table S2 (Supporting Information).

### Yeast One‐Hybrid Assays, Dual‐Luciferase Assay, Electrophoresis Mobility Shift Assay (EMSA)

For yeast one‐hybrid assays, the 1500 bp upstream the ATG codon of *SlAVT6A* and *SlAVT6B* were amplified and cloned into pHis2 vector. The full‐length coding sequence of SlWRKY57 was amplified and cloned into pGADT7. For dual‐luciferase assay, the promoter of *SlAVT6A* and *SlAVT6B* was cloned into the modified pH2GW7.0 vector to drive the firefly LUC reporter gene. The full‐length coding sequence of SlWRKY57 was amplified by PCR and cloned into the vector pEAQ.^[^ ^60^ ^]^ For EMSA, MBP‐SlWRKY57 proteins were expressed in *Escherichia coli* and purified using MBP Sepharose. The DNA probes of SlAVT6A and SlAVT6B were labeled with 5′FAM (fluorescein isothiocyanate) fluorescent dye (Synthesized by Tsingke Biotechnology, Beijing, China). All experiments were conducted as previously described.^[^ ^53^ ^]^

### Yeast Two‐Hybrid Assay, Luciferase Complementation (LCI) Assays, BiFC Assays

The interaction between the SlAVT6A and SlAVT6B was performed using the yeast split‐ubiquitin system. The CDS of *SlAVT6A* and *SlAVT6B* were cloned into the pBT3‐STE or pPR3‐N, respectively. The recombinant constructs were co‐transformed into yeast strain NMY51 and the transformed yeast cells were incubated on SD/‐Trp‐Leu medium for 3 days at 30 °C. The transformed yeast cells were then transferred to the SD/‐Trp‐Leu/‐His/‐Ade medium and incubated for 3 days. For LCI assay, *SlAVT6A* and *SlAVT6B* were cloned into the pCAMBIA1300‐cLUC and pCAMBIA1300‐nLUC vector, respectively. *N. benthamiana* leaves were infiltrated, then treated with 1 mm luciferin and visualized. For BiFC assay, the coding sequence of *SlAVT6A* and *SlAVT6B* were fused with C‐terminal or N‐terminal fragment of YFP. After co‐expression for 48 h, the leaves were imaged with a confocal laser scanning microscope to visualize yellow fluorescent protein fluorescence.

The interaction between the SlWRKY57 and SlJAZ8 was performed using the Y2H Gold‐Gal4 system (Clontech, http://www.clontech.com). Full‐length CDS of *SlJAZ8* and *SlWRKY57* were cloned into the pGADT7 or pGBKT7, respectively. The recombinant constructs were co‐transformed into yeast strain Y2H Gold and the transformed yeast cells were incubated on SD/‐Trp‐Leu medium for 3 days at 30 °C. The transformed yeast cells were then transferred to the SD/‐Trp‐Leu/‐His/‐Ade medium and incubated for 3 days. For LCI assay, *SlJAZ8* and *SlWRKY57* were cloned into the pCAMBIA1300‐cLUC and pCAMBIA1300‐nLUC vector, respectively.

### Preparation of Plant Extracts and Metabolite Analysis

The amino acid contents were measured using a liquid chromatography‐tandem mass spectrometry (LC‐MS/MS) system. 0.1 g powder was extracted with 1 mL of 70% aqueous methanol containing 0.1 mg L^−1^ lidocaine (internal standard) for overnight at 4 °C. Then, samples were centrifuged at 10 000 g for 10 min, and the soluble extracts were filtered (SCAA‐104, 0.22 µm pore size, ANPEL) before LC‐MS analysis. The samples were analyzed using a high‐resolution Thermo Scientific QExactive Plus hybrid quadrupole‐Orbitrap high‐resolution mass spectrometry to identify metabolites structures based on retention time, precursor ion (Q1), and production fragmentation information. Relative quantification of amino acid was carried out using a scheduled multiple reaction monitoring (sMRM) via UPLC‐ESI‐MS/MS‐QTRAP (AB Sciex QTRAP 6500+). The column was an ACQUITY UPLC HSS T3 (1.8 µm, 2.1 mm × 100 mm) from Waters Corporation (Milford, MA, U.S.A). The mobile phase was water (0.04% acetic acid in water, v/v) (mobile phase A) and acetonitrile (0.04% acetic acid in acetonitrile, v/v) (mobile phase B). The linear gradient of mobile phase B was 5–95% within 0–10 min, 95% within 10–11 min, and 95–5% within 11–11.1 min, 5% within 11.1–15 min, with a flow rate of 0.35 mL × min^−1^. The samples (2 µL) were injected into the system and analyzed in the positive electrospray ionization (ESI) mode. Detailed retention time and characteristic fragment ions for identification of amino acid were provided in Table S3 (Supporting Information).

Analysis of terpene was performed as described by,^[^ ^61^ ^]^ with minor modifications. The leaflets were harvested from the 6‐week‐old tomato plants and then immersed in 2 mL of methyl tertiary‐butyl ether (MTBE) containing 10 ng µL^−1^ naphthalene internal standard. Following a 5 min incubation period with gentle shaking at room temperature, the leaf was removed and its dry weight was determined. The extract solution was then analyzed by gas chromatography‐mass with the 7890B GC System (Agilent). The GC program used an injector temperature of 280 °C. The initial column temperature was held at 40 °C for 1 min and then ramped at 40 °C min^−1^ to 90 °C, 15 °C min^−1^ to 110 °C, 25 °C min^−1^ to 250 °C, and finally at 40 °C min^−1^ to 320 °C, which was maintained for 2 min. The helium carrier gas flow was set to 0.4 mL min^−1^.

The amount of gibberellin in wildtype and transgenic plants were determined according the method as described previously,^[^ ^62^ ^]^ full descriptions of analysis are included in Methods S1 (Supporting Information).

The content of IPP were measured based on previously methods, full descriptions of analysis are included in Methods S2 (Supporting Information).

### Spider Mite Bioassays

The preference assays, fecundity assays and inoculation assay of spider mites were performed as described previously,^[^ ^17^, ^18^ ^]^ full descriptions are included in Methods S3 (Supporting Information).

### Immunoblot Analysis of Protein Abundance

Protein was extracted from 5 weeks old *N. benthamiana* leaves transiently expressing SlWRKY57‐GFP treated with or without 100 µm MeJA for 12 h using RIPA buffer (Beyotime Biotechnology, China). After quantified protein concentration using Bradford solution, 50 µg total protein was separated by 10% SDS‐PAGE gel and immunoblotted with rabbit anti‐GFP (Abcam, ab290, 1:5000 dilution) or rabbit anti‐actin (Agrisera, AS132640, 1:5000 dilution) as the primary antibody and anti‐HRP (ABclonal, AS014, 1:5000 dilution) as the secondary antibody.

### Statistical Analysis

Statistical analyzes were performed using Origin software (https://www.originlab.com/origin). The details of the tests used are included in the figure legends.

## Conflict of Interest

The authors declare no conflict of interest.

## Author Contributions

S.W. and J.Y. conceived the project and supervised this study. Y.H., X.W., L.G., L.X., and E.L. performed the experiments. P.C., P.L., and Y.Z. participated in the material preparation. C.L. collected and analyzed the data. J.L. carried out the metabolite analyses. Y.H., S.W. and J.Y. wrote the original manuscript. All of the authors discussed the results and commented on the manuscript.

## Supporting information



Supporting Information

Supporting Information

## Data Availability

The data that support the findings of this study are available in the supplementary material of this article.

## References

[1] H. Yang , Y. Li , Y. Cao , W. Shi , E. Xie , N. Mu , G. Du , Y. Shen , D. Tang , Z. Cheng , Nat. Commun. 2022, 13, 485. 35079011 10.1038/s41467-022-28173-3 PMC8789853

[2] Y. He , J. Cheng , Y. He , B. Yang , Y. Cheng , C. Yang , H. Zhang , Z. Wang , Plant Biotechnol. J. 2019, 17, 322. 29947463 10.1111/pbi.12979 PMC6335077

[3] M. Tegeder , D. Rentsch , Mol. Plant 2010, 3, 997. 21081651 10.1093/mp/ssq047

[4] B. Heinemann , T. M. Hildebrandt , J. Exp. Bot. 2021, 72, 4634. 33993299 10.1093/jxb/erab182

[5] J. Cai , A. Aharoni , Curr. Opin. Plant Biol. 2022, 69, 102288. 35987012 10.1016/j.pbi.2022.102288

[6] E. Martinoia , S. Meyer , A. D. Angeli , R. Nagy , Annu. Rev. Plant Biol. 2012, 63, 183. 22404463 10.1146/annurev-arplant-042811-105608

[7] P. Dhatterwal , S. Mehrotra , A. J. Miller , R. Mehrotra , Plant Mol. Biol. 2021, 107, 451. 34674117 10.1007/s11103-021-01193-1

[8] S. Ogasawara , M. Ezaki , R. Ishida , K. Sueyoshi , S. Saito , Y. Hiradate , T. Kudo , M. Obara , S. Kojima , N. Uozumi , K. Tanemura , T. Hayakawa , Plant J. 2021, 107, 1616. 34216173 10.1111/tpj.15403

[9] Y. Fujiki , H. Teshima , S. Kashiwao , M. Kawano‐Kawada , Y. Ohsumi , Y. Kakinuma , T. Sekito , FEBS Lett. 2017, 591, 5. 27925655 10.1002/1873-3468.12507

[10] B.‐F. Zhu , L. Si , Z. Wang , Y. Z. Jingjie Zhu , Y. Shangguan , D. Lu , D. Fan , C. Li , H. Lin , Q. Qian , T. Sang , B. Zhou , Y. Minobe , B. Han , Plant Physiol. 2011, 155, 1301. 21263038 10.1104/pp.110.168500 PMC3046587

[11] Y. Xiao , H. Zhang , Z. Li , T. Huang , T. Akihiro , J. Xu , H. Xu , F. Lin , Plant Biotechnol. J. 2022, 20, 1888. 35678495 10.1111/pbi.13869 PMC9491460

[12] C. J. Snowden , B. Thomas , C. J. Baxter , J. A. C. Smith , L. J. Sweetlove , Plant J. 2015, 81, 651. 25602029 10.1111/tpj.12766 PMC4950293

[13] M. Wu , Q. Zhang , Y. Dong , Z. Wang , W. Zhan , Z. Ke , S. Li , L. He , S. Ruf , R. Bock , J. Zhang , New Phytologist 2022, 237, 1363. 36328788 10.1111/nph.18595

[14] J. Xu , Z. O. Herwijnen , D. B. Dräger , C. Sui , M. A. Haring , R. C. Schuurink , The Plant Cell 2018, 30, 2988. 30518626 10.1105/tpc.18.00571 PMC6354261

[15] Y. Zhang , H. J. Bouwmeester , I. F. Kappers , J. Exp. Bot. 2020, 71, 330. 31557301 10.1093/jxb/erz422 PMC6913709

[16] M. Liu , G. Hong , H. Li , X. Bing , Y. Chen , X. Jing , J. Gershenzon , Y. Lou , I. T. Baldwin , R. Li , Proc. Natl. Acad. Sci., U. S. A. 2023, 120, 2305007120. 10.1073/pnas.2305007120 PMC10266023 37256931

[17] Y. He , Y. Zhao , J. Hu , L. Wang , L. Li , X. Zhang , Z. Zhou , L. Chen , H. Wang , J. Wang , G. Hong , Mol. Plant 2024, 17, 258. 38069474 10.1016/j.molp.2023.12.003

[18] Q. Guo , I. T. Major , G. Kapali , G. A. Howe , New phytologist 2022, 236, 132. 35642375 10.1111/nph.18293 PMC9541860

[19] H. Yuan , W. Liu , Y. Lu , Cell Host Microbe 2017, 21, 143. 28182949 10.1016/j.chom.2017.01.007

[20] K. Ament , M. R. Kant , M. W. Sabelis , M. A. Haring , R. C. Schuurink , Plant Physiol. 2004, 135, 2025. 15310835 10.1104/pp.104.048694 PMC520773

[21] Y. Yuan , X. Xu , Y. Luo , Z. Gong , X. Hu , M. Wu , Y. Liu , F. Yan , X. Zhang , W. Zhang , Y. Tang , B. Feng , Z. Li , C.‐Z. Jiang , W. Deng , Plant Biotechnol. J. 2021, 19, 138. 32654333 10.1111/pbi.13448 PMC7769234

[22] B. Hua , J. Chang , M. Wu , Z. Xu , F. Zhang , M. Yang , H. Xu , L.‐J. Wang , X.‐Y. Chen , S. Wu , Plant Biotechnology Journal 2021, 19, 375. 32888338 10.1111/pbi.13473 PMC7868972

[23] Z. Gong , Y. Luo , W. Zhang , W. Jian , L. Zhang , X. Gao , X. Hu , Y. Yuan , M. Wu , X. Xu , X. Zheng , G. Wu , Z. Li , Z. Li , W. Deng , J. Exp. Bot. 2021, 72, 3806. 33619530 10.1093/jxb/erab086

[24] G. Zhu , S. Wang , Z. Huang , S. Zhang , Q. Liao , C. Zhang , T. Lin , M. Qin , M. Peng , C. Yang , X. Cao , X. Han , X. Wang , E. van der Knaap , Z. Zhang , X. Cui , H. Klee , A. R. Fernie , J. Luo , S. Huang , Cell 2018, 172, 249 . 29328914 10.1016/j.cell.2017.12.019

[25] J. Tone , A. Yamanaka , K. Manabe , N. Murao , M. Kawano‐Kawada , T. Sekito , Y. Kakinuma , Biosci., Biotechnol., Biochem. 2015, 79, 190. 25266154 10.1080/09168451.2014.963501

[26] J. Besnard , R. Pratelli , C. Zhao , U. Sonawala , E. Collakova , G. Pilot , S. Okumoto , J. Exp. Bot. 2016, 67, 6385. 27856708 10.1093/jxb/erw412 PMC5181585

[27] O. Sakiko , K. Wolfgang , T. Mechthild , F. Wolf , B. Alexander , L. Dario , D. S. York , F. Wolf , J. Exp. Bot. 2004, 55, 2155.15361541

[28] H. Lei , J. Ma , S. S. Martinez , T. Gonen , Nat. Struct. Mol. Biol. 2018, 25, 522. 29872228 10.1038/s41594-018-0072-2 PMC7346717

[29] K. Junsu , P. Hahnbeom , H. Lim , S. Chaok , Nucleic Acids Res. 2012, 40, W294.22649060

[30] W. Tian , C. Chen , X. Lei , J. Zhao , J. Liang , Nucleic Acids Res. 2018, 46, W363. 29860391 10.1093/nar/gky473 PMC6031066

[31] J. Glas , B. Schimmel , J. Alba , R. Escobar‐Bravo , R. Schuurink , M. Kant , Int. J. Mol. Sci. 2012, 13, 17077. 23235331 10.3390/ijms131217077 PMC3546740

[32] Y. Chen , D. Su , J. Li , S. Ying , H. Deng , X. He , Y. Zhu , Y. Li , Y. Chen , J. Pirrello , M. Bouzayen , Y. Liu , M. Liu , J. Exp. Bot. 2020, 71, 3450. 32133496 10.1093/jxb/eraa114 PMC7475245

[33] W. Tan , Q. Han , Y. Li , F. Yang , J. Li , P. Li , X. Xu , H. Lin , D. Zhang , New Phytologist 2021, 231, 1220. 33904185 10.1111/nph.17422

[34] K. Sato , M. Yamane , N. Yamaji , H. Kanamori , A. Tagiri , J. G. Schwerdt , G. B. Fincher , T. Matsumoto , K. Takeda , T. Komatsuda , et al., Nat. Commun. 2016, 7, 11625. 27188711 10.1038/ncomms11625 PMC4873977

[35] Y. Jiang , D. Yu , Plant Physiol. 2016, 171, 2771. 27268959 10.1104/pp.16.00747 PMC4972294

[36] Y. Jiang , G. Liang , S. Yang , D. Yu , Plant Cell 2014, 26, 230. 24424094 10.1105/tpc.113.117838 PMC3963572

[37] J. Ma , C. Li , L. Sun , X. Ma , H. Qiao , W. Zhao , R. Yang , S. Song , S. Wang , H. Huang , J. Int. Plant Biol. 2023, 65, 2437. 10.1111/jipb.13562 37665103

[38] N. Guo , J. Hu , M. Yan , H. Qu , L. Luo , M. Tegeder , G. Xu , Plant J. 2020, 103, 395. 32159895 10.1111/tpj.14742

[39] H. Svennerstam , U. Ganeteg , T. Näsholm , New Phytologist 2008, 180, 620. 18681934 10.1111/j.1469-8137.2008.02589.x

[40] L. Zhang , Q. Tan , R. Lee , A. Trethewy , Y.‐H. Lee , M. Tegeder , Plant Cell 2010, 22, 3603. 21075769 10.1105/tpc.110.073833 PMC3015121

[41] M. Tegeder , U. Z. Hammes , Curr. Opin. Plant Biol. 2018, 43, 16. 29278790 10.1016/j.pbi.2017.12.002

[42] N. Guo , D. Xue , W. Zhang , J.‐M. Zhao , C.‐C. Xue , Q. Yan , J.‐Y. Xue , H.‐T. Wang , Y.‐M. Zhang , H. Xing , J. Int. Agr. 2016, 15, 1727.

[43] N. Su , A. Zhu , X. Tao , Z. J. Ding , S. Chang , F. Ye , Y. Zhang , C. Zhao , Q. Chen , J. Wang , C. Zhou , Y. Guo , S. Jiao , S. Zhang , H. Wen , L. Ma , S. Ye , S. J. Zheng , F. Yang , S. Wu , Ji. Guo , Nature 2022, 609, 616. 35917926 10.1038/s41586-022-05142-w

[44] L. Wang , K. Chen , M. Zhou , Nat. Commun. 2021, 12, 4455. 34294705 10.1038/s41467-021-24778-2 PMC8298490

[45] H. E. McFarlane , J. J. H. Shin , D. A. Bird , A. L. Samuels , Plant Cell 2010, 22, 3066. 20870961 10.1105/tpc.110.077974 PMC2965547

[46] J. H. Lynch , I. Orlova , C. Zhao , L. Guo , R. Jaini , H. Maeda , T. Akhtar , J. Cruz‐Lebron , D. Rhodes , J. Morgan , G. Pilot , E. Pichersky , N. Dudareva , The Plant J. 2017, 92, 939. 28977710 10.1111/tpj.13730

[47] J. M. Alba , B. C. J. Schimmel , J. J. Glas , L. M. S. Ataide , M. L. Pappas , C. A. Villarroel , R. C. Schuurink , M. W. Sabelis , M. R. Kant , New Phytologist 2015, 205, 828. 25297722 10.1111/nph.13075 PMC4301184

[48] Y. Yang , S. Cai , Y. Zou , H. Ai , Z. Zou , T. Xin , B. Xia , Z. Zou , Crop Protection 2024, 176, 106470.

[49] A. Kessler , I. T. Baldwin , Science 2001, 291, 2141. 11251117 10.1126/science.291.5511.2141

[50] C. Schnee , T. G. Köllner , M. Held , T. C. Turlings , J. Gershenzon , J. Degenhardt , Proc. Natl. Acad. Sci., U. S. A. 2006, 103, 1129. 16418295 10.1073/pnas.0508027103 PMC1347987

[51] I. Maoz , E. Lewinsohn , I. Gonda , Curr. Opin. Plant Biol. 2022, 67, 102221. 35533493 10.1016/j.pbi.2022.102221

[52] L. Wang , G. Xu , L. Li , M. Ruan , A. Bennion , G.‐L. Wang , R. Li , S. Qu , Proc. Natl. Acad. Sci., U. S. A. 2023, 120, 2211102120. 10.1073/pnas.2211102120 PMC10068787 36952381

[53] Y. Hao , L. Xiang , J. Lai , C. Li , Y. Zhong , W. Ye , J. Yang , J. Yang , S. Wang , New Phytologist 2023, 239, 1353. 37287391 10.1111/nph.19048

[54] G. Hu , K. Wang , B. Huang , I. Mila , P. Frasse , E. Maza , A. Djari , M. Hernould , M. Zouine , Z. Li , M. Bouzayen , Nat. Plants 2022, 8, 419. 35422080 10.1038/s41477-022-01121-1

[55] H. Kang , J. H. Sul , S. Service , N. Zaitlen , S. Kong , N. Freimer , C. Sabatti , E. Eskin , Nat. Genet. 2010, 42, 348. 20208533 10.1038/ng.548 PMC3092069

[56] M.‐X. Li , J. M. Y. Yeung , S. S. Cherny , P. C. Sham , Human Genetics 2012, 131, 747. 22143225 10.1007/s00439-011-1118-2 PMC3325408

[57] C. Sun , L. Deng , M. Du , J. Zhao , Q. Chen , T. Huang , H. Jiang , C.‐B. Li , C. Li , Mol. Plant 2020, 13, 42. 31678614 10.1016/j.molp.2019.10.010

[58] M. Du , Q. Zhai , L. Deng , S. Li , H. Li , L. Yan , Z. Huang , B. Wang , H. Jiang , T. Huang , C. Li , J. Wei , L. Kang , J. Li , C. Li , The Plant Cell 2014, 26, 3167. 25005917 10.1105/tpc.114.128272 PMC4145139

[59] H. Guo , M. Mao , Y. Deng , L. Sun , R. Chen , P. Cao , J. Lai , Y. Zhang , C. Wang , C. Li , Y. Li , Q. Bai , T. Tan , J. Yang , S. Wang , Frontiers in Plant Science 2022, 13, 860577. 35463452 10.3389/fpls.2022.860577 PMC9024245

[60] B. Hua , J. Chang , M. Wu , Z. Xu , F. Zhang , M. Yang , H. Xu , L.‐J. Wang , X.‐Y. Chen , S. Wu , Plant Biotechnol. J. 2021, 19, 375. 32888338 10.1111/pbi.13473 PMC7868972

[61] J.‐H. Kang , F. Shi , A. D. Jones , M. D. Marks , G. A. Howe , J. Exp. Bot. 2009, 61, 1053. 20018901 10.1093/jxb/erp370 PMC2826649

[62] L. V. Meulebroek , J. Bussche , K. Steppe , L. Vanhaecke , J. Chromatogr. A 2012, 1260, 67. 22980641 10.1016/j.chroma.2012.08.047

