# Supplementary material for: JA‐Mediated Regulation of Amino Acid Homeostasis Adjusts Metabolic Flux and Enhances Spider Mite Tolerance via the SlJAZ8‐SlWRKY57‐SlAVT6s Module in Tomato
Source: Adv Sci (Weinh). 2025 Jun 10;12(31):e16717. doi: 10.1002/advs.202416717 (PMC12376665; doi:10.1002/advs.202416717)
Supplement: Supplementary file 1 — Supporting Information [file ADVS-12-e16717-s001.pdf]

ADVANCED SCIENCE
Open Access

Supporting Information

for *Adv. Sci.*, DOI 10.1002/advs.202416717

JA-Mediated Regulation of Amino Acid Homeostasis Adjusts Metabolic Flux and Enhances Spider Mite Tolerance via the SlJAZ8-SlWRKY57-SlAVT6s Module in Tomato

*Yingchen Hao, Xiaolong Wang, Langchen Guo, Lijun Xiang, Enxi Luo, Peng Cao, Penghui Liu, Yue Zhong, Chun Li, Jun Lai, Jun Yang and Shouchuang Wang**

Supplementary Materials

JA-mediated regulation of amino acid homeostasis adjusts metabolic flux and enhances spider mite tolerance via the SlJAZ8-SlWRKY57-SlAVT6s module in tomato

Authors: Yingchen Hao, Xiaolong Wang, Langchen Guo, Lijun Xiang, Enxi Luo, Peng Cao, Penghui Liu, Yue Zhong, Chun Li, Jun Lai, Jun Yang, Shouchuang Wang

The following Supporting Information is available for this article:

Figure S1 Frequencies of the high/low-level trait-related allele of SlAVT6B and SlAVT6A in three groups.
Figure S2 Subcellular localization of SlAVT6A and SlAVT6B in *N. benthamiana* leaves.
Figure S3 Transformants of SIAVT6A, SIAVT6B and AtAAP3 grown on plates treated with 10mM NH_4^+ or 2mM amino acid for 3 days.
Figure S4 Pairwise query-template sequence alignments used for structural model predictions.
Figure S5 The 3D structure of SlAVT6A-SlAVT6B heterodimer. The box is enlarged to show the interaction of SlAVT6A and SlAVT6B.
Figure S6 Construction of the *SlAVT6A* and *SlAVT6B* transgenic tomato.
Figure S7 The internodal length, stem diameters and stem-leaf angle of WT, *SlAVT6A* and *SlAVT6B* transgenic plants
Figure S8 The densities of trichomes on the stem, and sepals of WT, *SlAVT6A* and *SlAVT6B* transgenic plants.
Figure S9 The infected area in WT, *SlAVT6A* and *SlAVT6B* plants after inoculation with spider mites.
Figure S10 The amino acid concentration in WT, *SlAVT6A* and *SlAVT6B* plants.
Figure S11 Effect of SlAVT6A and SlAVT6B on the expression of MVA (a) and MEP (b) metabolic genes.
Figure S12 Effect of SlAVT6A and SlAVT6B on the expression of GA biosynthesis genes.
Figure S13 The trichome phenotypes abundance on the leaf of WT, *SlAVT6A* and *SlAVT6B* plants under PAC treatment.
Figure S14 Construction of *SlWRKY57* transgenic lines.
Figure S15 SlWRKY57 affects the trichome density in tomato.
Figure S16 The quantification of α-Thujene and β-Pinene in WT and *SlWRKY57* plants.
Figure S17 Knockdown of *SlWRKY57* enhances spider mite resistance in tomato.
Figure S18 SlAVT6A and SlAVT6B affect the nitrogen assimilation.
Figure S19 Yield trait analysis of WT, *SlAVT6A* and *SlAVT6B* plants.
Methods S1 Metabolite analysis of GA in tomato leaves.
Methods S2 Metabolite analysis of IPP, FPP and GGPP in tomato leaves.
Methods S3 Spider mite bioassays.
Note: Supplementary Tables S1-S6 are provided as separate files.

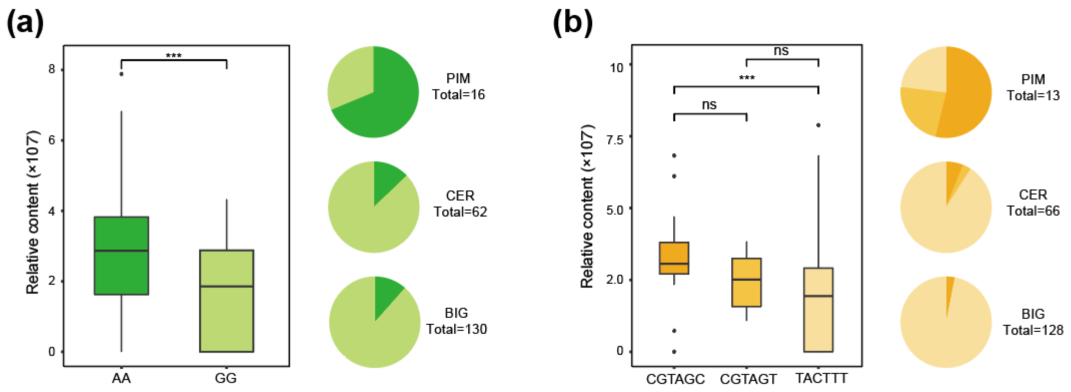

Figure S1 Frequencies of the high/low-level trait-related allele of SlAVT6B (a) and SlAVT6A (b) in three groups.

Box plot indicates the relative contents of phenylalanine. The metabolic data was log2 transformed. Data are presented as mean ± SD. *P*-values are calculated using t-test, *** *P*<0.001.

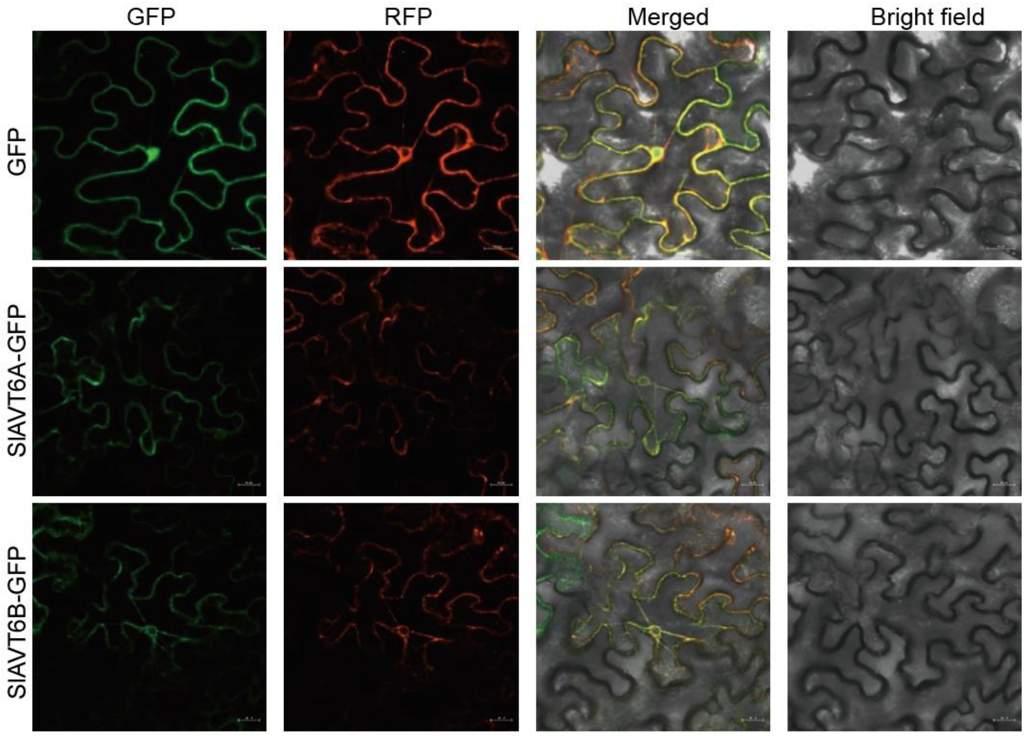

Figure S2 Subcellular localization of SlAVT6A and SlAVT6B in *N. benthamiana* leaves. Tobacco leaves were transiently transformed with empty GFP vector, SlAVT6A-GFP and SlAVT6B-GFP construct via *A. tumefaciens transfection*. A fluorescence microscope was used to observe GFP fluorescence. AT1G73190-RFP [1] was used as vacuole positive control. Scale bars = 20 μm.

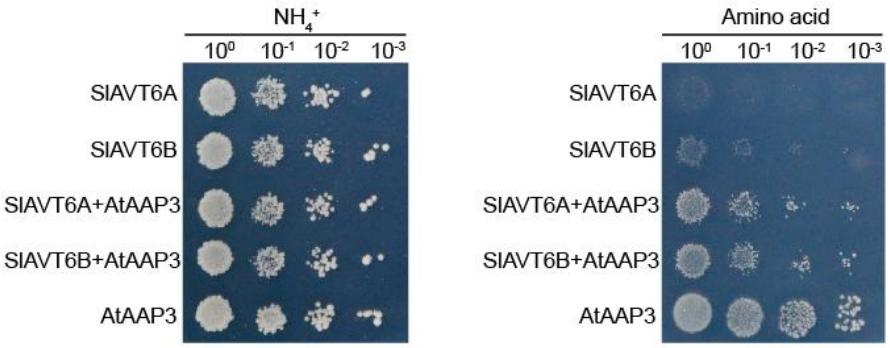

Figure S3 Transformants of SIAVT6A, SIAVT6B and AtAAP3 grown on plates treated with 10mM NH_4^+ or 2mM amino acid for 3 days.

Serial dilutions (10^0, 10^{-1}, 10^{-2}, 10^{-3}) of yeast transformants were plated onto media for further screening.

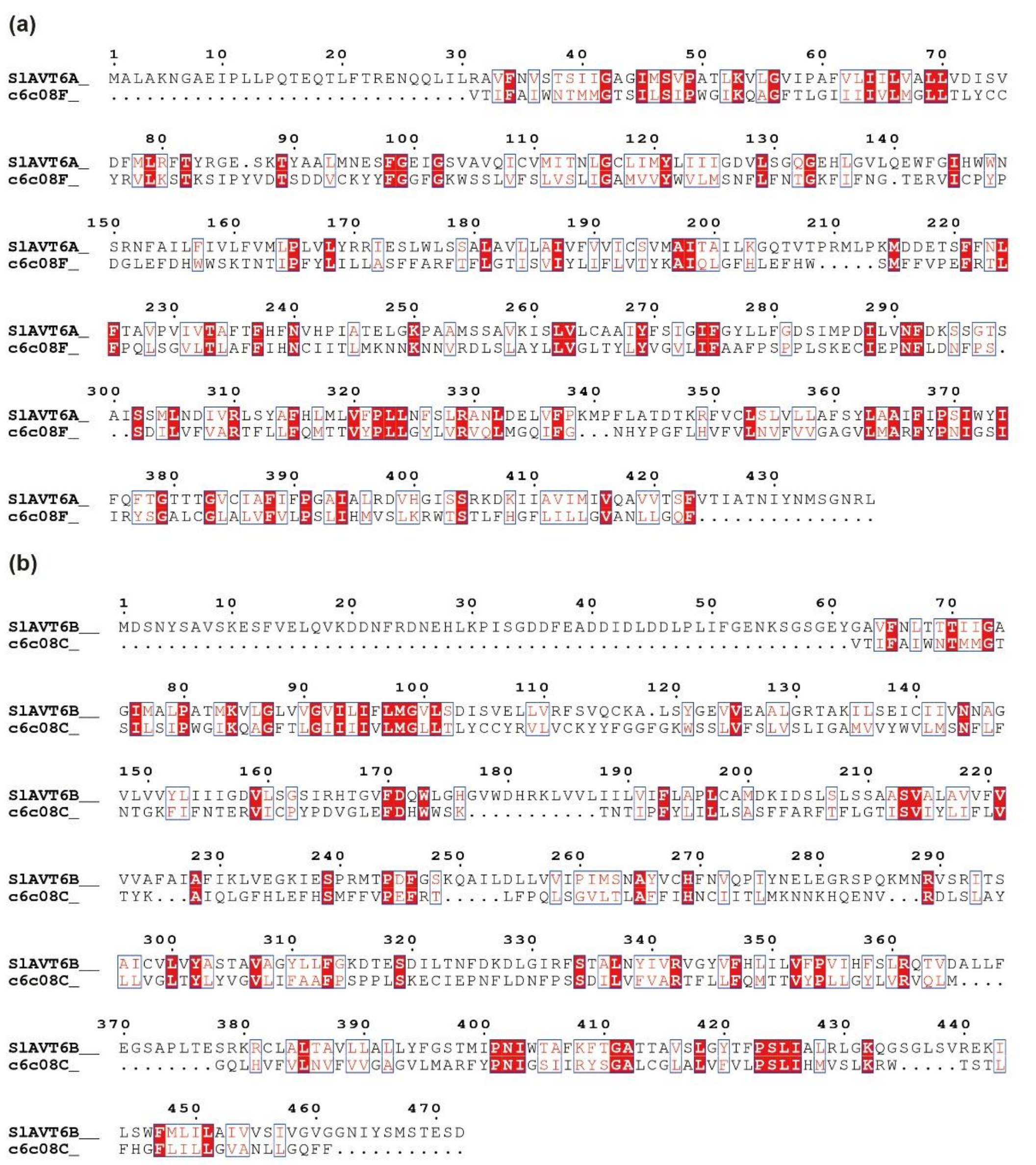

Figure S4 Pairwise query-template sequence alignments used for structural model predictions. (a) Structural alignments and predicted secondary structures for SlAVT6A using sodium-coupled neutral amino acid transporter 9 (PDB c608F) as template. (b) Alignments and secondary structure predictions for SlAVT6B using sodium-coupled neutral amino acid transporter 9 (PDB c608C). Alignments obtained in protein were generated by CLUSTALW and visualized with ESPript 3.0.

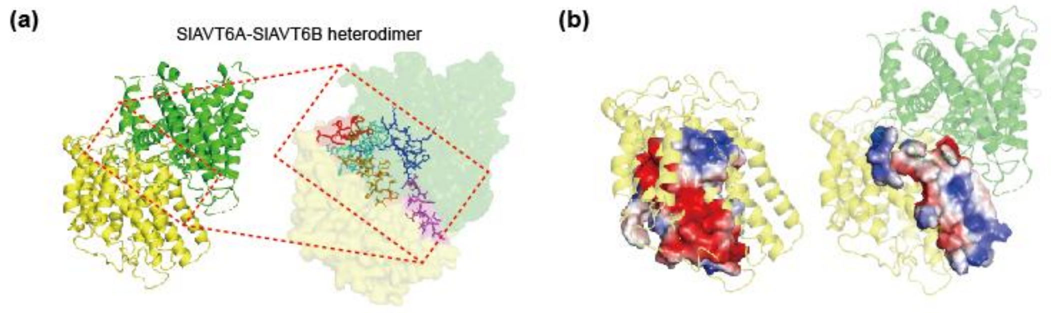

Figure S5 The 3D structure of SlAVT6A-SlAVT6B heterodimer. The box is enlarged to show the interaction of SlAVT6A and SlAVT6B.

(a) Proposed active pocket of SlAVT6A. (b) Proposed active pocket of the SlAVT6A-SlAVT6B heterodimer. Direct interactions are indicated by different colors.

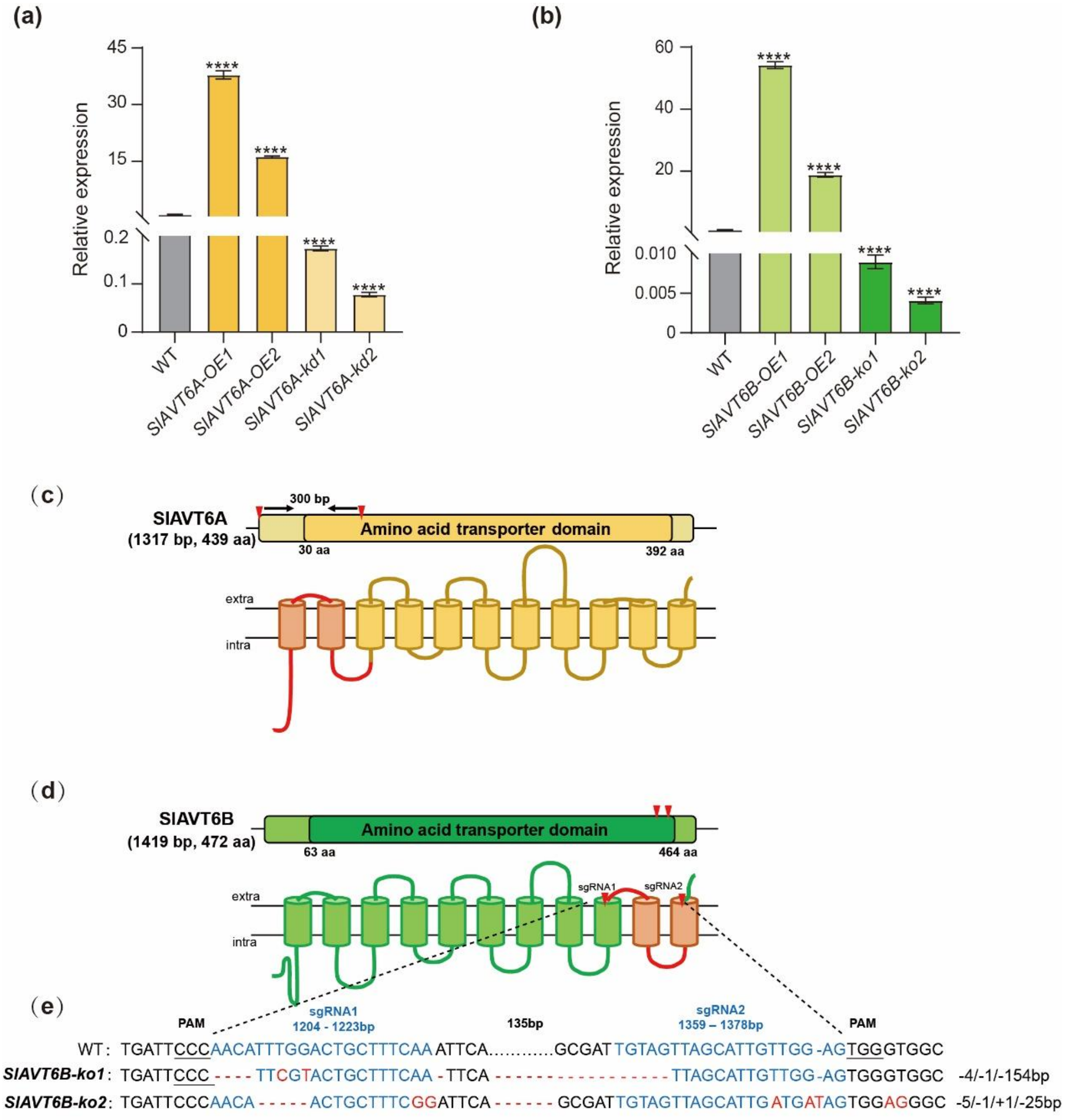

Figure S6 Construction of the *SlAVT6A* and *SlAVT6B* transgenic tomato.

(a-b) expression levels of *SlAVT6A* or *SlAVT6B* in transgenic plants. The data are represented as mean ± SD, n = 3, *P*-values are calculated using t-test, **** *P*<0.0001. (c) Gene structure and transmembrane domain of SlAVT6A. The target sequences of SlAVT6A via RNA interference (RNAi) mechanism are indicated by black arrows and highlighted in red. (d) Gene structure and transmembrane domain of SlAVT6B. The target sequences by two sgRNAs are highlighted in the red. (e) Nucleotide sequences of the regions encompassing the target sequences in the *SlAVT6B-ko* lines.

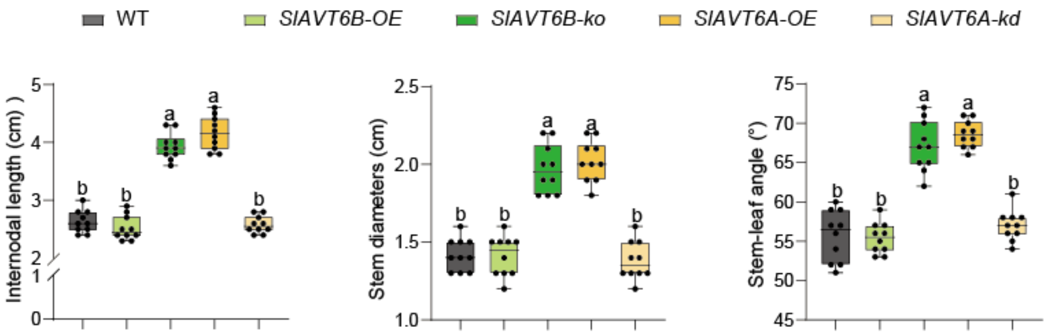

Figure S7 The internodal length, stem diameters and stem-leaf angle of WT, SlAVT6A and SlAVT6B transgenic plants.

Data are represented as means ± SD, n = 10, *P*-values are calculated using Bonferroni test after ANOVA, significance was defined as $P \leq 0.05$.

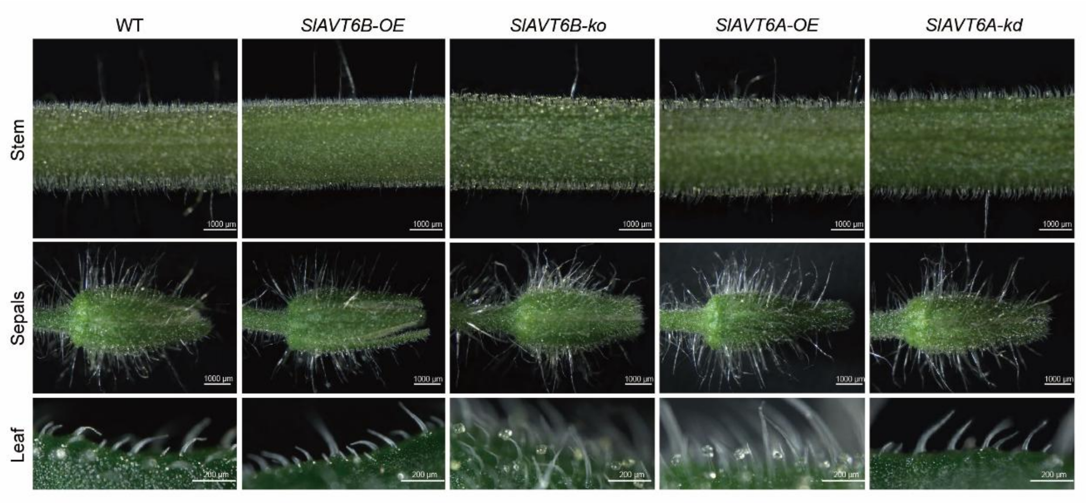

Figure S8 The densities of trichomes on the stem, and sepals of WT, *SlAVT6A* and *SlAVT6B* transgenic plants.

6-week-old plants were used for all photographs. Scale bar = 2000 µm.

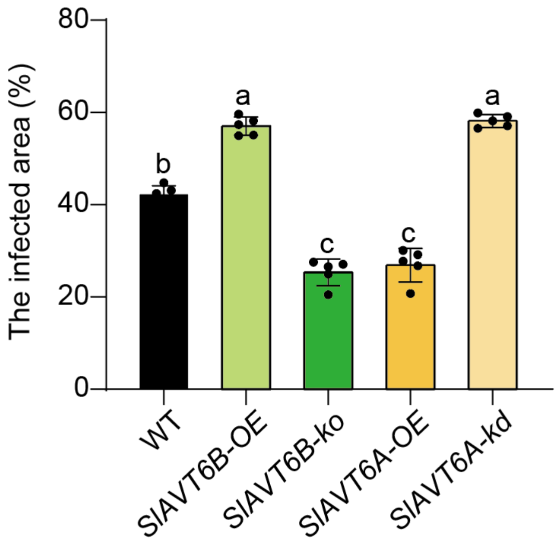

Figure S9 The infected area in WT, *SlAVT6A* and *SlAVT6B* plants after inoculation with spider mites for 45 days.

Data are represented as means ± SD, n = 5, *P*-values are calculated using Bonferroni test after ANOVA, significance was defined as *P*≤0.05.

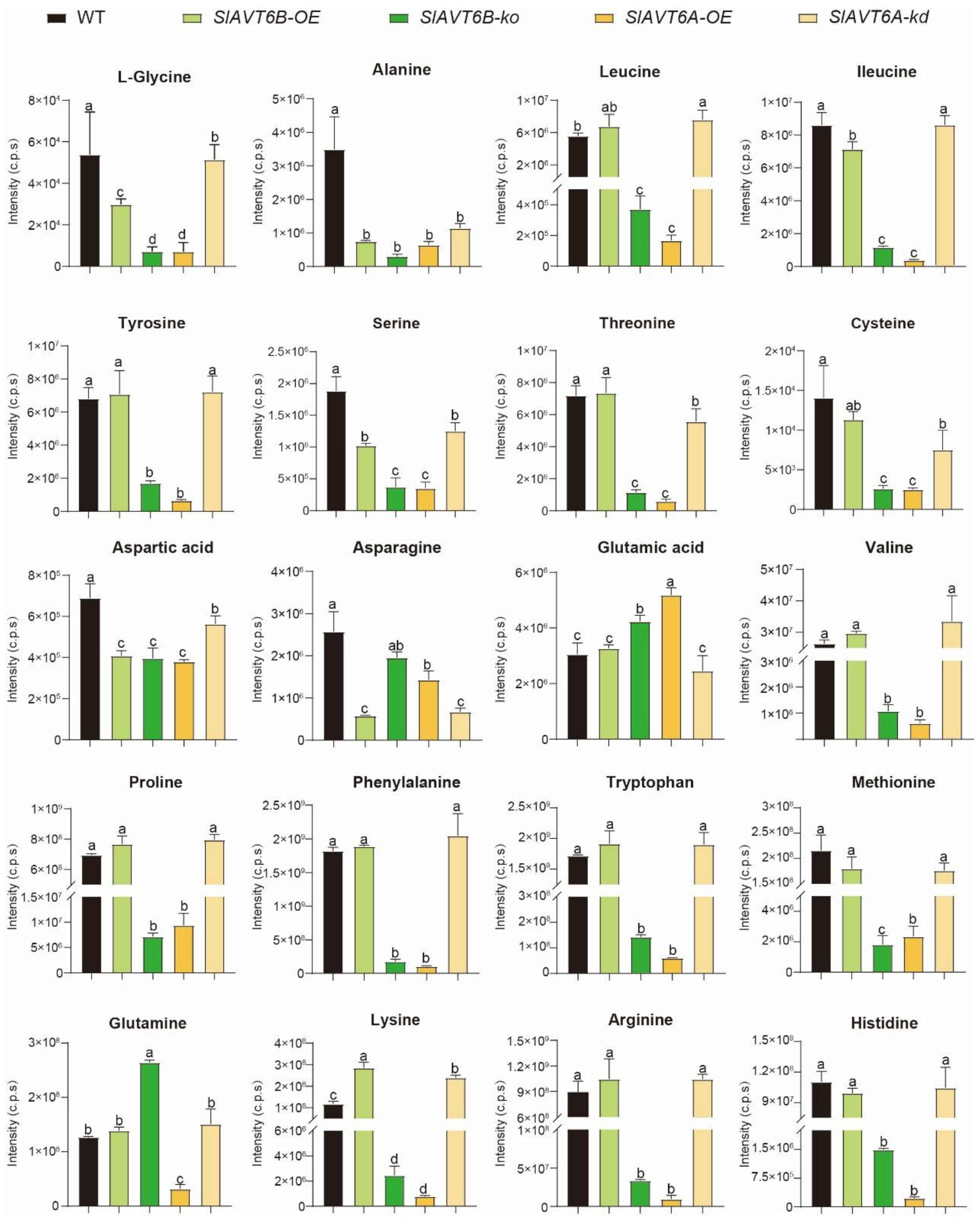

Figure S10 The amino acid concentration in WT, *SlAVT6A* and *SlAVT6B* plants.

Data are represented as means ± SD, n = 3), *P*-values are calculated using Bonferroni test after ANOVA, significance was defined as $P \leq 0.05$.

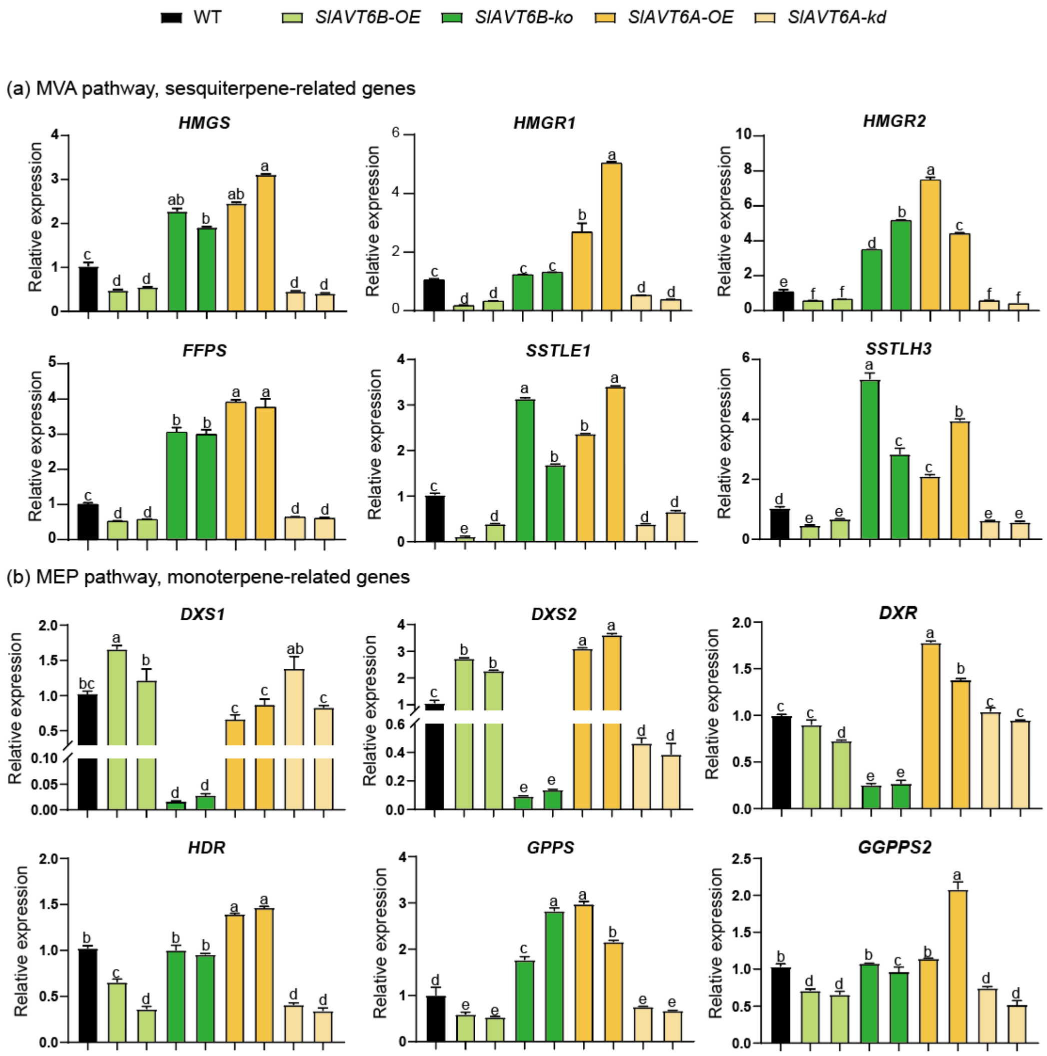

Figure S11 Effect of SlAVT6A and SlAVT6B on the expression of MVA (a) and MEP (b) metabolic genes.

Data are represented as means ± SD, n = 3, *P*-values are calculated using Bonferroni test after ANOVA, significance was defined as $P \leq 0.05$.

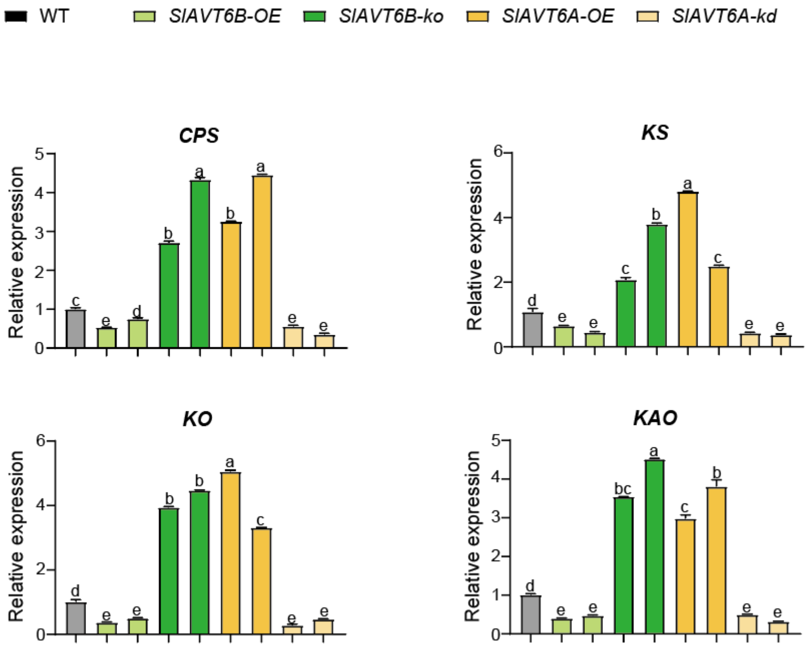

Figure S12 Effect of SlAVT6A and SlAVT6B on the expression of GA biosynthesis genes.

Data are represented as means ± SD, n = 3, *P*-values are calculated using Bonferroni test after ANOVA, significance was defined as $P \leq 0.05$.

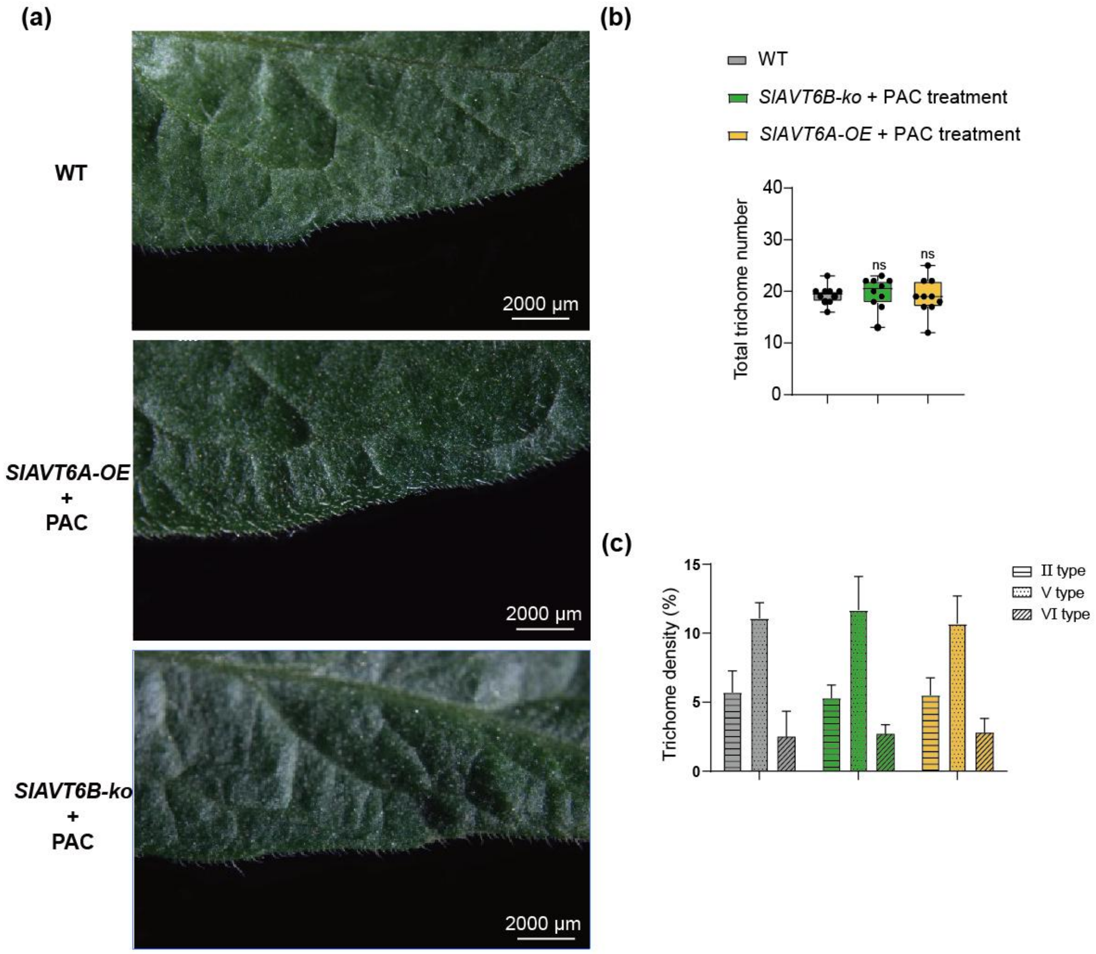

Figure S13 The trichome phenotypes of *SlAVT6A* and *SlAVT6B* plants under PAC treatment. (a) The phenotypes of trichome abundance on the leaf of WT, SlAVT6A and SlAVT6B plants under PAC treatment. 6-week-old plants were used for all photographs. Scale bar = 2000 μm. (b) The total trichome number of WT, SlAVT6A and SlAVT6B plants under PAC treatment. Data are represented as means ± SD, n = 10, *P*-values are calculated using t-test. (c) The trichome density of WT, SlAVT6A and SlAVT6B plants under PAC treatment. Data are represented as means ± SD, n = 10.

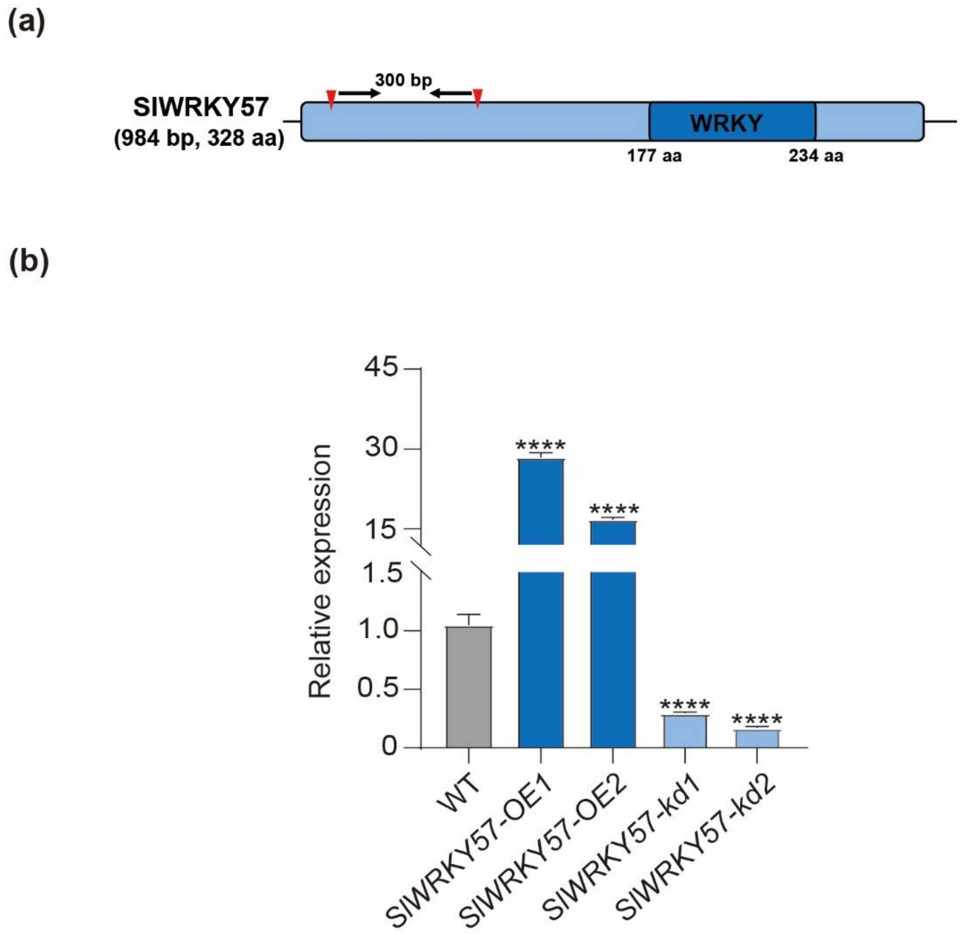

Figure S14 Construction of *SlWRKY57* transgenic lines.

(a) The target sequences of *SlWRKY57-kd* via RNA interference (RNAi) mechanism. (b) The expression levels of *SlWRKY57* in WT and *SlWRKY57* transgenic lines. The data are represented as mean ± SD, n = 3, *P*-values are calculated using t-test, **** *P*<0.0001.

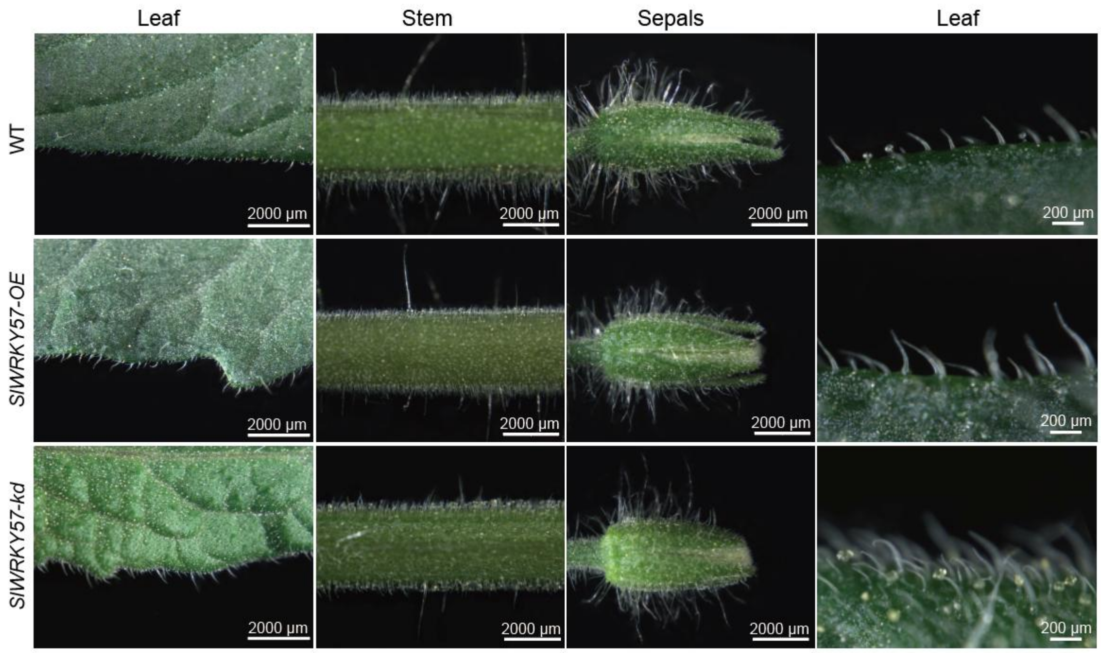

Figure S15 SlWRKY57 affects the trichome density in tomato.

The densities of trichomes on the leaves, stem, and sepals of *SlWRKY57* transgenic plants. 6-week-old plants were used for all photographs. Scale bar = 2000 μm or 200 μm.

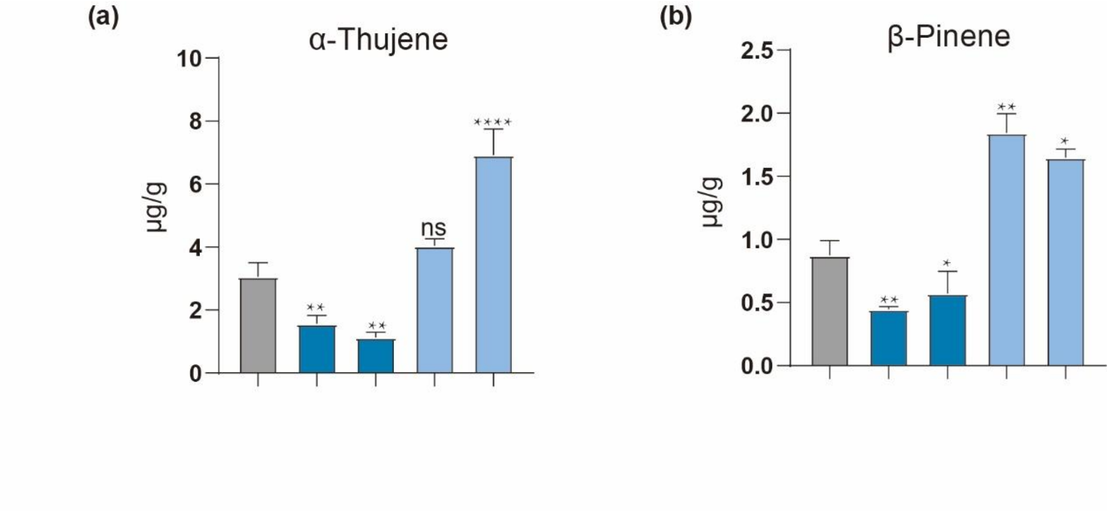

Figure S16 The quantification of α-Thujene and β-Pinenein in WT and *SlWRKY57* plants.

The Y-axis represents the content of the terpenes (μg) per gram (g) of the fresh leaf. The data represent means ± SD, n = 3, *P*-values are calculated using t -test, * *P*<0.05; ** *P*<0.01; *** *P*<0.001; **** *P*<0.0001.

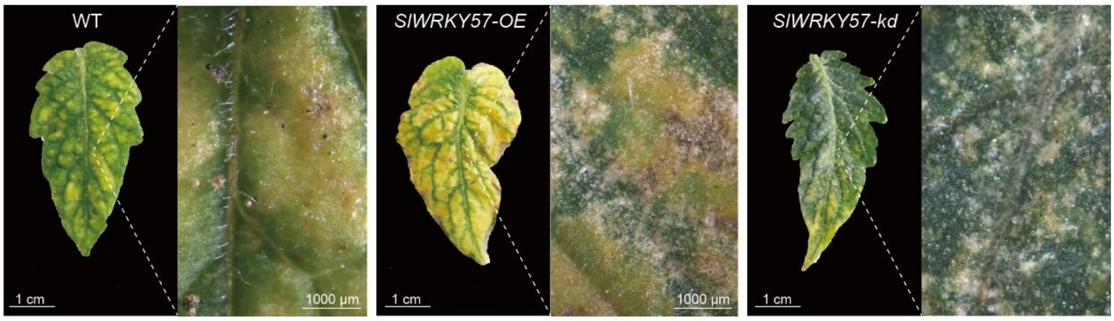

Figure S17 Knockdown of *SlWRKY57* enhances spider mite resistance in tomato.

Inoculation of WT and *SlWRKY57* transgenic plants with spider mites for 45 days. Fifteen adult female mites were transferred to a single leaf on 45-day-old WT and *SlWRKY57* transgenic plants. Scale bars = 1000 μm.

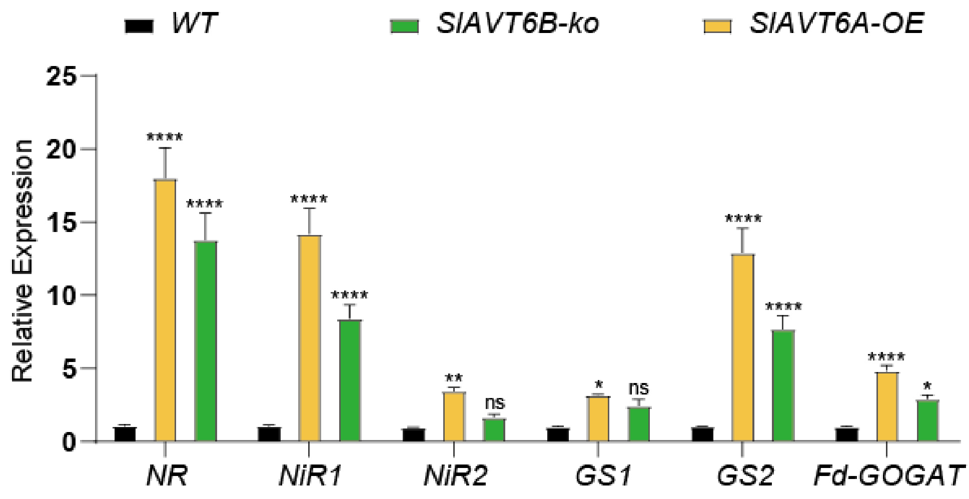

Figure S18 SlAVT6A and SlAVT6B affect the nitrogen assimilation.

Comparison of nitrogen assimilation genes expression in WT, *SlAVT6A-OE* and *SlAVT6B-ko* using qRT-PCR. The expression of genes was normalized to that of SlActin gene control. Data are represented as means ± SD, n = 3, *P*-values are calculated using t-test, * *P*<0.05; ** *P*<0.01; *** *P*<0.001; **** *P*<0.0001.

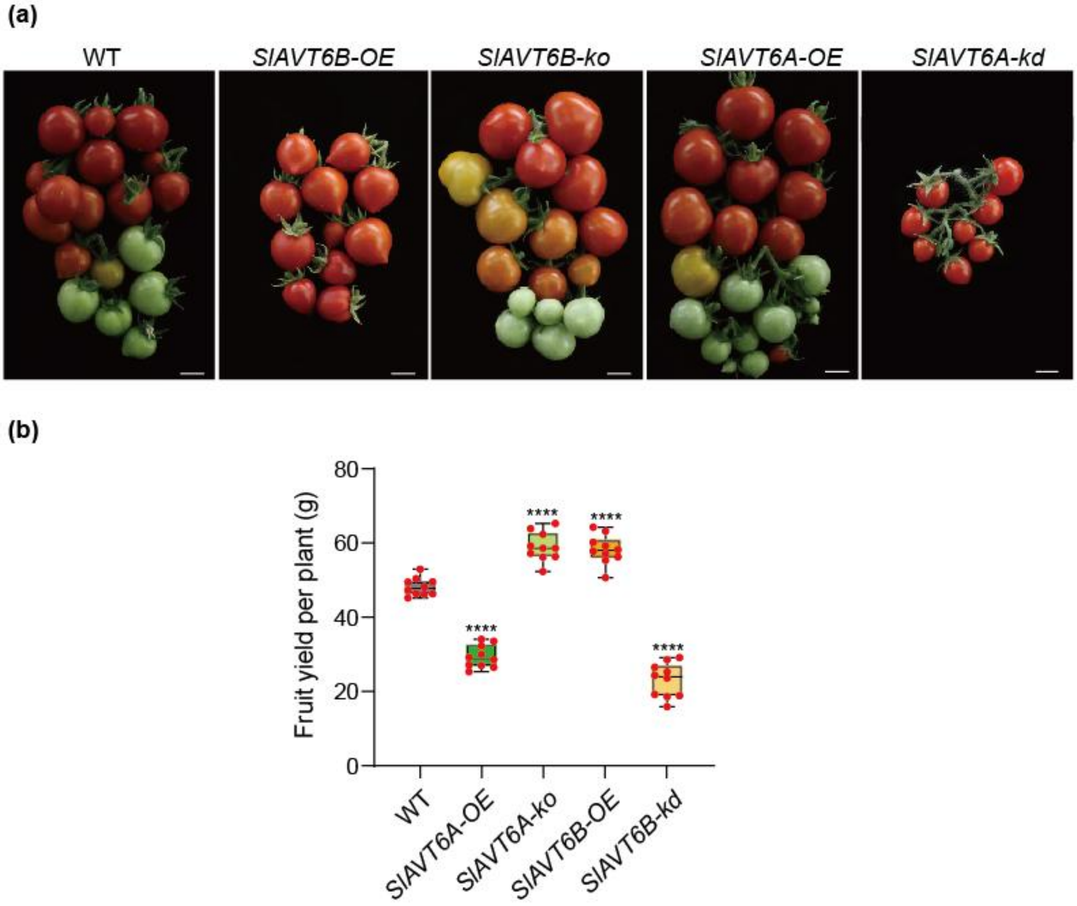

Figure S19 Yield trait analysis of WT, *SlAVT6A* and *SlAVT6B* plants.

(a) Increased fruit number per plant in *SlAVT6B-ko* and *SlAVT6A-OE* plants. Scale bar = 1 cm. (b) Total fruit yield determined by assessing the total fruit weight per plant in plants. Values are represented as means ± SD, n = 10, *P*-values are calculated using t-test, **** $P<0.0001$.

Methods 1 Metabolite analysis of GA in tomato leaves

100 mg of ground sample was mixed with an extraction solution containing methanol-water-formic acid (75:20:5) at a ratio of 1:10,000 (w/v). The mixture was subjected to ice-bath ultrasonication for 30 minutes and then left at -20°C for 16 hours. After centrifugation at 12,000 rpm for 10 minutes, the supernatant was collected. The extraction was repeated on the pellet, and the supernatants were combined and filtered. Solid-phase extraction (SPE) was subsequently performed: the SPE column was activated sequentially with methanol and pure water, equilibrated, and then loaded in aliquots (⩽1 mL per loading). Elution was carried out using 80% methanol, and the loading and elution fractions were pooled. Finally, the combined solution was concentrated to dryness using nitrogen evaporation, reconstituted in 80% methanol to a final volume of 100 μL, filtered, and stored in a brown autosampler vial with an insert. The mobile phase was 5 mM ammonium bicarbonate aqueous solution (mobile phase A) and acetonitrile (mobile phase B). The mobile phase was water (0.04% acetic acid in water, v/v) (mobile phase A) and acetonitrile (0.04% acetic acid in acetonitrile, v/v) (mobile phase B). The linear gradient of mobile phase B was 5%-95% within 0-10 min, 95% within 10-11 min, and 95%-5% within 11-11.1 min, 5% within 11.1-15 min, with a flow rate of 0.35 ml × min-1. The samples (2 μL) were injected into the system and analyzed in the positive electrospray ionization (ESI) mode.

Methods 2 Metabolite analysis of IPP, FPP and GGPP in tomato leaves

0.5 g of lyophilized tomato leaf samples were extracted with 500 μL of extraction solution (methanol: ammonia =70:30, v/v). The samples were ultrasonic in ice water for 30 min. Then, samples were centrifuged at 10,000 g for 10 min, and the soluble extracts were used for LC-MS analysis. The samples were analyzed by AB Sciex 7500 Qtrap, a highly sensitive and low-resolution LC-MS. Relative quantification of IPP, FPP and GGPP was carried out using a scheduled multiple reaction monitoring (sMRM). The column was shim-pack VP-ODS C18 (1.9 μm, 2.1 x 100 mm) from Shimadzu (Shimadzu, Japan). The liquid phase conditions were as follows: the sample size was 5 μL. The mobile phase was 5 mM ammonium bicarbonate aqueous solution (mobile phase A) and acetonitrile (mobile phase B). The linear gradient of mobile phase B was 5% to 20% within 0-2 min, 20% to 100% within 2-6 min, 100% maintain for 1 min, 100% to 5% within 0.1 min, 5% for 12 min. The flow rate was 0.35 mL × min^{-1}, and the column temperature was 25°C. The data was analyzed using OS software (Sciex). Detailed retention time and characteristic fragment ions for identification of IPP, FPP and GGPP were provided in Table S3.

Methods 3 Spider mite bioassays.

To assess the resistance of tomato plants to spider mites, we conducted spider mite bioassays across three dimensions: spider mites preference assays, oviposition assays, and whole-plant infestation assays. In the preference assay, 10 healthy mites were placed equidistantly between 6-week-old transgenic and wild-type plants at room temperature for two hours, after which the number of mites transferred to the leaves of transgenic and wild-type plants was recorded. For the oviposition assay, leaves of similar developmental stages from 6-week-old transgenic and wild-type plants were placed on moist filter paper. Five healthy female mites were transferred onto the leaves, and the number of eggs laid on the leaves was counted every 24 hours. In the whole-plant infestation assay, 15 healthy female mites were transferred onto the third leaf of 6-week-old transgenic and wild-type plants of comparable size and growth status. After two and four weeks of cultivation, the plant phenotypes were observed, and photographs were taken.

Reference

[1] Z. Mao, W. Sun, J. Exp. Bot. 2015, 66 4781.
